# Biodiversity of *Trichoderma* Community in the Tidal Flats and Wetland of Southeastern China

**DOI:** 10.1371/journal.pone.0168020

**Published:** 2016-12-21

**Authors:** Kandasamy Saravanakumar, Chuanjin Yu, Kai Dou, Meng Wang, Yaqian Li, Jie Chen

**Affiliations:** 1 School of Agriculture and Biology, Shanghai Jiao Tong University, Shanghai, P.R. China; 2 State Key Laboratory of Microbial Metabolism, Shanghai Jiao Tong University, Shanghai, P.R. China; 3 Key Laboratory of Urban Agriculture (South), Ministry of Agriculture, Shanghai, P.R. China; National University of Ireland—Galway, IRELAND

## Abstract

To investigate the biodiversity of *Trichoderma* (*Hypocreaceae*) and their relation to sediment physical and chemical properties, we collected a total of 491 sediment samples from coastal wetlands (tidal flat and wetland) in Southeast China. Further, we applied two types of molecular approaches such as culture dependent and independent methods for identification of *Trichoderma* spp. A total of 254 isolates were obtained and identified to 13 species such as *T*. *aureoviride*, *T*. *asperellum*, *T*. *harzianum*, *T*. *atroviride*, *T*. *koningiopsis*, *T*. *longibrachiatum*, *T*. *koningii*. *T*. *tawa*, *T*. *viridescens*, *T*. *virens*, *T*. *hamatum*, *T*. *viride*, and *T*. *velutinum* by the culture-dependent (CD) method of these, *T*. *tawa* was newly described in China. Subsequently, the culture indepented method of 454 pyrosequencing analysis revealed a total of six species such as *T*. *citrinoviride*, *T*. *virens*, *T*. *polysporum*, *T*. *harzianum*/*Hypocrea lixii* and two unknown species. Notably, *T*. *citrinoviride* and *T*. *polysporum* were not found by the CD method. Therefore, this work revealed that the combination of these two methods could show the higher biodiversity of *Trichoderma* spp., than either of this method alone. Among the sampling sites, Hangzhou, Zhejiang Province, exhibited rich biodiversity and low in Fengxian. Correlation and Redundancy discriminant analysis (RDA) revealed that sediment properties of temperature, redox potential (Eh) and pH significantly influenced the biodiversity of *Trichoderma* spp.

## Introduction

*Trichoderma* speceis are economically important fungi, occurs in various environments at different geographical locations [1–4]. *Trichoderma* research have been received a global interest because of their biotechnological and agricultural applications. Especially, *Trichoderma* strains are extensively used as biocontrol agents to reduce plants infections caused by phytopathogens [5]. Biodiversity of *Trichoderma* in China is richly diversified because of varied ecological conditions [6]. Several microbial ecologists have been investigated *Trichoderma* biodiversity in various geographical locations in China [7].

According to Zhu and Zhuang [8], a total of 91 *Trichoderma* spp., have been reported from various ecological samples collected from China, such as agriculture substrate [7, 9–14] rhizosphere sediment [15], and edible fungi [16–17]. In which, most of strains are derived from agriculture resources and fewer from other resources. Moreover, researchers have mostly studied the biodiversity of *Trichoderma* spp., by using microscopic examination followed by sequencing of the internal transcribed spacer regions (ITS) and/or partial translation elongation factor-1α (*tef*-1) [3,7,18], but these methods cannot provide the information about culture independent strains due to close relationship between *Trichoderma* sp., [19]. Moreover, the accurate identification of *Trichoderma* spp., based on the morphological method is difficult because of morphological similarly between the species [20, 21] and that can be identified only based on the multiple gene sequences of DNA characters [22, 23]. Molecular-based multiple genes sequence [rDNA and genes encoding actin, calmodulin, endochitinase, RNA polymerase II, and translation elongation factor 1-alpha (*tef*-α)] analysis could offer the consistent identification of *Trichoderma* spp., [20, 24, 25]. To the best of our knowledge, there is no report on the biodiversity of *Trichoderma* in coastal wetlands in China and also there is no comparison studies on culture-dependent and independent methods in assessment of *Trichoderma* biodiversity. Therefore, in this work, we studied the biodiversity of *Trichoderma* spp., in coastal wetland through both culture-dependent and independent methods. For traditional molecular methods (culture-dependent), we used the two-genes approach of internal transcribed spacer regions 1 and 2 (ITS1 and ITS2) followed by translation-elongation factor 1-alpha (*tef*-α) [7,19] and culture independent method were used the 454 pyrosequencing technique. In addition, we analyzed the relation of culture-dependent and independent *Trichoderma* counts on sediment properties of coastal wetlands in Southeast China.

## Materials and Methods

### Ethics statement

No specific permissions were required for these locations/activities for native research institutes or researchers. In addition, this study was funded by Chinese government for the enrichment of public welfare and also this study did not involve endangered or protected species.

### Description of study area and sample collection

The study area is located in the Southeast China coast of Yellow Sea (Yantai, Shandong; Lianyungang, Jiangsu), East Sea (Huangpu River, Fengxian, Chongming Shanghai; Hangzhou, Ningbo Zhejiang; Fuzhou, Fujian), South Sea (Shantou, Zhuhai, Guangdong), North Sea (Beihai Guangxi) [S1 Table]. A total of 491 sediment samples were collected from different coastal wetlands at two seasons of spring and summer, 2014. Prior to collect the sediment samples the sampling site was marked equally for 10 m x 10 m dimension (quadrate plot of 10 m^2^) using a nylon rope and GPS reading was recorded and it was divided into four sub-sample plots (5 m x 5 m). At least one sample was taken from each sub-sample plot at 5 different sediment depths (0–20, 20–40, 40–60, 60–80 and 80–100 cm) by using a corer (1.5 m long stainless steel corer with 50 mm diameter) during low tide and made one composite sample according to the depth respectively. The composite of sediment were placed in pre-cleaned polyethylene bags and immediately transported to laboratory and stored at -20°C for CD microbiological and pyrosequencing analysis.

### *In situ* and laboratory sediment properties analysis

The sediment temperature, Eh, and pH were determined at the time of sampling. The temperature was measured using a thermometer with ±0.5°C accuracy. Hydrogen ion concentration of the sediment sample was measured using a pH meter with a platinum electrode with an accuracy of ± 0.1, (pH 315i/ SET, Wissenschaftlich Technische Werkstatten, Germany) and calibrated with standard buffer solution prior to use. Porewater salinity was recorded by using a hand refractometer (Atago hand refractometer, Japan), after crushing a small amount of sediment through Whatman No.1 filter paper with a syringe. For this, a known amount of sediment sample was moisturized with double distilled water up to the moisture saturation level of the sediment.

In order to analysis the sediment properties by the laboratory experiments, the sediment samples were separately collected and immediately transferred to the laboratory in sterile polyethene bags. Plant roots, and other debris were removed from the samples and dried in an oven at 110°C and ground to fine powder. Sediment composition of clay, silt and sand were analyzed using a hydrometer method [26]. Total organic carbon in sediment was estimated by adopting the method of El Wakeel and Riley [27].

### Culture-depentent isolation of *Trichoderma* spp.

The soil dilution method was adopted for isolation of the *Trichoderma* by using a Modified Potato- dextrose agar (PDAm) [28]. In detail, 1 g of sediment sample was serially diluted and one milliliter (10^4^ dilution factor) of the serially diluted sample was pipetted out into sterile PDAm Petri-dishes (90 x 15 mm) and spread over the surface of the PDAm by an L-shaped glass rod and incubated at 25±2°C for 5 to 7 days. After the incubation, the *Trichoderma* colonies were enumerated and calculated the *Trichoderma* load in the given sample using the standard formula for Colony Forming Unit (CFU) per gram of the sample. All the determinations were carried out in triplicate.

### Identification of culture-dependent *Trichoderma* spp.

*Trichoderma* isolates were identified at the species level by using the combination of morphological [29–32] and microbial molecular analysis [7]. The isolates were purified by repeated sub-culturing and the pure cultures were stored by cryopreservation in 20% glycerol at -80°C in School of Agriculture and Biology, Shanghai Jiao Tong University, P. R. China.

### Genomic DNA sequence-based identification

Genomic DNA was extracted from mycelium of *Trichoderma* pure cultures grown on potato dextrose agar (PDA) for 3 d according to the method of Doohan et al [33]. The ITS 1 and ITS 2 regions of the rDNA gene were amplified using the ITS4 and ITS5 primers [34]. Followed by a fragment of the partial translation elongation factor 1-alpha (*tef*-α) gene was amplified using the primer EF728M [35] and tef1R [36]. The 50 μl reaction mixture consisted of 2 μl 50 ng/μl of template DNA, 25μl of 2x *Taq* PCR MasterMix with loading dye (Tiangen, China), 0.2 μl 100 mM of each primer, and 19 μl of MQ H_2_O. Polymerase chain reactions (PCR) were carried out under conditions described by Błaszczyk et al. [19]. Further, the PCR products were separated in 1.2% of agarose gel as described by Sun et al. [7].

Finally, 3 μl of the PCR products were purified with exonuclease I and shrimp alkaline phosphatase [37]. The sequences were analyzed by with a MegaBACE 1000 DNA automatic sequencing system (Pharmacia), with the DYEnamic ET Dye Terminator Cycle sequencing kit (Pharmacia). The obtained sequences were edited using Chromas V.1.43 (Applied Pharmacia). The successfully sequenced ITS and *tef1*-α genes were aligned using the CLUSTAL W Multiple Sequence Alignment Program [38].

For molecular species identification, ITS sequences were submitted to BLAST interface analysis in NCBI (http://blast.ncbi.nlm.gov/) and *Tricho*OKey (http://www.isth.info) [20, 39]. In ambiguous cases, the result was rechecked using NCBI (http://blast.ncbi.nlm.gov/) and the *Tricho*BLAST program based on *tef*1-α gene sequences [40,41].

Phylogenetic trees and molecular evolutionary analysis were made using the MEGA version 6.0 [42]. The phylogenetic tree was constructed according to the neighbor-joining, maximum parsimony, and maximum likelihood algorithms [43, 44]. In order to evaluate the consensus of the branching, the bootstrap of phylogenetic tree analysis was employed with 1000 replicates of the data set. A suite of SPSS 11.5 software (IBM) was used for the statistical analysis to find the relation between culture depentent *Trichoderma* spp., counts (CFU) and other sediment properties (Sediment depths, sites or seasons) by ANOVA followed by post hoc test (Tukey’s) and correlation analysis using Pearson’s correlation method. In addition to this, PRIMER (Plymouth Routines in Multivariate Ecological Research (version 6.1.10) was used to analyze the diversity indices.

### Culture independent *Trichoderma* spp., analysis

Genomic DNA was extracted from the sediment samples using the TIANamp soil DNA kit according to manufacturer’s instructions (Tiangen, China). After extraction, the purity of the DNA was tested using a UV spectrophotometer followed by 0.8% agarose gel electrophoresis at a voltage of 120 V for 20 min. The internal transcribed spacer (ITS) regions were amplified using forward primer: (5’-GGAAGTAAAAGTCGTAACAAGG-3’) and reverse primer: (5’-TCCTCCGCTTATTGATATGC-3’). PCR reaction was conducted in 20 ng/μl of reaction mixture consisted of 8.75 μl of ultra pure H_2_O, 5 μl of 5x Q5 Buffer, 5 μl of 5x GC Enhancer, 2 μl of dNTP (2.5mM), 2 μl of template DNA (2ng/ μl), 1 μl each forward and reverse primer (10 μM), 0.25 μl of Q5 DNA polymerase. PCR cycling conditions were set as 4 min at 98°C, 27 cycles (98°C for the 30s, 47.6°C for the 45s, and 72° C for the 1 min), and then the final extension at 72°C for 5 min, and the experiment was halted at 10°C. PCR products were purified using AMpure Beads followed by the PicoGreen dsDNA assay kit used for the quantification of DNA. The mixture was pyrosequenced by using Roche 454 GS FLX (Shanghai Personalbio Co., Ltd., China). The sequenced data was analyzed by using Qiime (version 1.7.0, http://qiime.org/) followed by version 1.31.2, http://www.mothur.org/ and further the data was analyzed according to Sun et al. [6]. In brief, the below sequences score of 25 and 200 bp length were trimmed and binned into operational taxonomic units (OTUs) using a 97% of the threshold for the bioinformatics and subsequent analysis [6]. Finally, the Redundancy discriminant analysis (RDA) of R-vegan and R-map tools for Linex were selected to find the relationship between the analyzed sediment environmental parameters and *Trichoderma* spp.

## Results

### Biodiversity of culture-depentent *Trichoderma* spp.

A total of 254 isolates of *Trichoderma* (S2 Table) were isolated and identified based on a combination of morphological, ecological, and phylogenetic gene sequences of ITS1, ITS2 and translation-elongation factor 1-alpha *(tef*1); the nucleotide sequences were deposited in NCBI GenBank database (Accession numbers: KR868229- KR868570). Out of 254 isolates, a total of 13 species were identified such as *T*. *aureoviride* Rifai (12 isolates), *T*. *asperellum* Samuels, Lieckf. & Nirenberg, Sydowia (32), *T*. *harzianum* Rifai (63), *T*. *atroviride* Bissett (134), *T*. *koningiopsis* Samuels, C. Suarez & H.C. Evans (2), *T*. *longibrachiatum* Rifai (4), *T*. *koningii* Oudamans (1). *T*. *tawa* P. Chaverri & Samuels (1), *T*. *viridescens* A.S. Horn & H.S. Will. Jaklitsch & samuels (1), *T*. *virens* J.H. Miller, Giddens & A.A. Foster Arx (1), *T*. *hamatum* Bonord (1), *T*. *viride* Pers (1), and *T*. *velutinum* Bissett, C.P. Kubicek & Szakacs (1 isolate). The identification, origin, and NCBI accession numbers of the strains are given in Table 1.

**Table 1:** Regional representative of *Trichoderma* spp., reported in this study.

Culture collection	Isolation source/ location	Representative isolate name	Organism identification	NCBI accession number		Clade
				ITS1,ITS2	*tef1-α*	
CCTCC-SBW0007	Soil,wetland forest, Chongming, Shanghai	CHI1	*Trichoderma asperellum*	KR868230	KR868401	Viride
CCTCC-SBW0108	Rhizosphere soil, Mangroves-Avicennia alba zone Zhuhai	ZHMT9	*Trichoderma harzianum*	KR868232	KR868403	Green/Harzianum
CCTCC-SBW0106	Rhizosphere soil, Mangroves- Avicennia marina zone, Zhuhai	ZHMT7	*Trichoderma asperellum*	KR868233	KR868404	Viride
CCTCC-SBW0105	Rhizosphere soil, Mangroves-Bruguiera gymnorrhiza, Zhuhai	ZHMT6	*Trichoderma asperellum*	KR868240	KR868411	Viride
CCTCC-SBW0114	Soil, Yinsha beach, Zhuhai	ZHYAQT2	*Trichoderma harzianum*	KR868244	KR868415	Green/Harzianum
CCTCC-SBW0097	Rhizosphere soil, Mangroves-Avicennia marina zone, Zhuhai	ZHMT14	*Trichoderma harzianum*	KR868245	KR868416	Green/Harzianum
CCTCC-SBW0022	Soil, Wetland east forest, Chongming, Shanghai	CHIPUFI2	*Trichoderma atroviride*	KR868247	KR868418	Viride
CCTCC-SBW0021	Soil, Wetland forest, Chongming, Shanghai	CHIA	*Trichoderma atroviride*	KR868248	KR868419	Viride
CCTCC-SBW0016	Soil, Wetland south forest, Chongming, Shanghai	CHI5	*Trichoderma atroviride*	KR868249	KR868420	Viride
CCTCC-SBW0023	Soil, Wetland forest, Chongming, Shanghai	PU2	*Trichoderma longibrachiatum*	KR868251	KR868422	Longibrachiatum
CCTCC-SBW0008	Soil, Wetland north forest, Chongming, Shanghai	CHI11	*Trichoderma atroviride*	KR868252	KR868423	Viride
CCTCC-SBW0011	Soil, Wetland forest, Chongming, Shanghai	CHI2	*Trichoderma asperellum*	KR868255	KR868426	Viride
CCTCC-SBW0024	Soil, Wetland forest, Chongming, Shanghai	PUFP1	*Trichoderma atroviride*	KR868256	KR868427	Viride
CCTCC-SBW0019	Soil, Wetland forest, Chongming, Shanghai	CHI8	*Trichoderma asperellum*	KR868259	KR868430	Viride
CCTCC-SBW0100	Rhizosphere soil, Mangroves-Avicennia marina zone Zhuhai	ZHMT21	*Trichoderma harzianum*	KR868260	KR868431	Green/Harzianum
CCTCC-SBW0054	Soil, wetland, Fuzhou	FJWT4	*Trichoderma asperellum*	KR868266	KR868437	Viride
CCTCC-SBW0052	Soil, wetland, Fuzhou	FJWT2	*Trichoderma asperellum*	KR868267	KR868438	Viride
CCTCC-SBW0058	Soil, wetland, Fuzhou	FJWT8	*Trichoderma asperellum*	KR868271	KR868442	Viride
CCTCC-SBW0053	Soil, wetland, Fuzhou	FJWT3	*Trichoderma asperellum*	KR868272	KR868443	Viride
CCTCC-SBW0080	Sediment, Wetland, Shantou	STWT4	*Trichoderma asperellum*	KR868273	KR868444	Viride
CCTCC-SBW0084	Sediment, Wetland, Shantou	STWT6	*Trichoderma asperellum*	KR868274	KR868445	Viride
CCTCC-RW0002	Soil Qian Tang River, Hangzhou	ZQTR1	*Trichoderma longibrachiatum*	KR868276	KR868447	Longibrachiatum
CCTCC-RW0023	Sediment, wetland park, Hangzhou	ZWPUEB14	*Trichoderma tawa*	KR868278	KR868449	Green/Harzianum
CCTCC-RW0017	Sediment, wetland park, Hangzhou	ZWPH1	*Trichoderma viridescens*	KR868281	KR868452	Viride
CCTCC-RW0003	Sediment, wetland park, Hangzhou	ZEPUEB7	*Trichoderma aureoviride*	KR868289	KR868460	Green
CCTCC-RW0007	Sediment, Botanical garden, wetland park, Hangzhou	ZWPBG2	*Trichoderma asperellum*	KR868290	KR868461	Viride
CCTCC-SBW0169	Coastal form soil, Ningbo, Zhejiang	ZNCF17	*Trichoderma harzianum*	KR868300	KR868471	Green/Harzianum
CCTCC-SBW0155	Beach water, Ningbo, Zhejiang	ZNBW11	*Trichoderma harzianum*	KR868303	KR868474	Green/Harzianum
CCTCC-SBW0102	Mangroves rhizosphere soil, Zhuhai	ZHMT4	*Trichoderma asperellum*	KR868308	KR868479	Viride
CCTCC-SBW0006	Soil, wetland forest, Chongming, Shanghai	CHI (WIN)	*Trichoderma harzianum*	KR868309	KR868480	Green/Harzianum
CCTCC-SBW0018	Soil, wetland forest, Chongming, Shanghai	CHI7	*Trichoderma atroviride*	KR868310	KR868481	Viride
CCTCC-SBW0104	Mangroves rhizosphere soil, Zhuhai	ZHMT5	*Trichoderma asperellum*	KR868312	KR868483	Viride
CCTCC-SBW0099	Mangroves rhizosphere soil, Zhuhai	ZHMT20	*Trichoderma harzianum*	KR868313	KR868484	Green/Harzianum
CCTCC-SBW0076	Wetland sediment, Shantou	STWT1	*Trichoderma longibrachiatum*	KR868317	KR868488	Longibrachiatum
CCTCC-SBW0088	water sample, wetland, Shantou	SW2	*Trichoderma velutinum*	KR868318	KR868489	Green/Harzianum
CCTCC-RW0009	Sediment, Botanical garden, wetland park, Hangzhou	ZWPBG4	*Trichoderma koningiopsis*	KR868323	KR868494	Viride
CCTCC-RW0010	Sediment, Botanical garden, wetland park, Hangzhou	ZWPBG5	*Trichoderma koningii*	KR868324	KR868495	Viride
CCTCC-SBW0051	Wetland soil, Fuzhou	FJWT14	*Trichoderma viride*	KR868325	KR868496	Viride
CCTCC-RW0028	Sediment, wetland park, Hangzhou	ZWPUEB4	*Trichoderma asperellum*	KR868326	KR868497	Viride
CCTCC-RW0025	Sediment, wetland park, Hangzhou	ZWPUEB2	*Trichoderma longibrachiatum*	KR868327	KR868498	Longibrachiatum
CCTCC-RW0016	Sediment, Botanical garden, wetland park, Hangzhou	ZWPBG9	*Trichoderma virens*	KR868328	KR868499	Green
CCTCC-SBW0136	Aquaculture form soil, Ningbo, Zhejiang	ZNAF20	*Trichoderma hamatum*	KR868332	KR868503	Viride
CCTCC-SBW0164	Beach water, Ningbo, Zhejiang	ZNBW8	*Trichoderma harzianum*	KR868343	KR868514	Green/Harzianum
CCTCC-SBW0175	Coastal form water, Ningbo, Zhejiang	ZNCFW3	*Trichoderma harzianum*	KR868348	KR868519	Green/Harzianum
CCTCC-SBW0163	Beach water, Ningbo, Zhejiang	ZNBW7	*Trichoderma harzianum*	KR868349	KR868520	Green/Harzianum
CCTCC-SBW0189	Harbor soil, Ningbo, Zhejiang	ZNH11	*Trichoderma aureoviride*	KR868365	KR868536	Green
CCTCC-SBW0160	Beach water, Ningbo, Zhejiang	ZNBW3	*Trichoderma harzianum*	KR868383	KR868554	Green/Harzianum
CCTCC-SBW0207	Reservoir soil, Ningbo, Zhejiang	ZNR20	*Trichoderma harzianum*	KR868390	KR868561	Green/Harzianum
CCTCC-SBW0180	Beach water, Ningbo, Zhejiang	ZNE3	*Trichoderma harzianum*	KR868394	KR868565	Green/Harzianum
CCTCC-SBW0225	Estuary soil, Ningbo, Zhejiang	ZNWPL9	*Trichoderma harzianum*	KR868395	KR868566	Green/Harzianum
CCTCC-SBW0161	Wetland soil, Ningbo, Zhejiang	ZNBW3	*Trichoderma harzianum*	KR868396	KR868567	Green/Harzianum
CCTCC-SBW0126	Aquaculture form soil, Ningbo, Zhejiang	ZNAF13	*Trichoderma atroviride*	KR868399	KR868570	Viride

*Trichoderma atroviride*, *T*. *harzianum* and *T*. *asperellum* were abundant in sediment samples than other *Trichoderma* spp. According to the recent report of Jaklitsch and Voglmayr [45], *Trichoderma* spp., were identified in this study belongs to four Clade, as follows: *Green* (13), *Green/Harzianum* (65), *Longibrachiatum* (4), and *Viride* (172). Species diversity was determined by diversity indices of Margalef *d* and Shannon-Wiener, *H’* (log*e*) analysis (Table 2). The *d* and *H’* in sampling stations ranged from 0–2.731 and 0–1.91 respectively and found higher in the Hangzhou Zhejiang Province and least in the Fengxian, Shanghai, Lianyungang, Jiangsu Province and Beihai, Guangxi Zhuang Autonomous region respectively. The distribution of *Trichoderma* spp., isolated from the different coastal wetlands was shown in the Table 3. The number of isolates was found high in Ningbo, Zhejiang province and less in Lianyungang, Fengxian, and Beihai. *Trichoderma atroviride* and *T*. *harzianum* counts were high in the coastal region of Ningbo, Zhejiang province. Hence, these results revealed that the coastal wetland ecosystem of Zhejiang province was richly diverse for *Trichoderma* spp.

**Table 2:** Univariate diversity indices analysis.

Sample	Diversity indices					
	S	N	D	J'	H'(loge)	1-Lambda'
Fengxian	1	1	-	-	0	-
Chongming	5	45	1.051	0.721	1.16	0.6131
Zhuhai	4	26	0.9208	0.8467	1.174	0.6862
Shantou	6	16	1.803	0.8038	1.44	0.7333
Beihai	1	3	0	-	0	0
Fuzhou	4	17	1.059	0.889	1.232	0.7279
Hangzhou	10	27	2.731	0.8293	1.91	0.8319
Ningbo	4	108	0.6407	0.5968	0.8273	0.4848
Lianyungang	1	8	0	-	0	0
Yantai	4	4	2.164	1	1.386	1

S–Number of species; N-Number of individuals; Values in parenthesis, Diversity constants species richness- Margalef, *d*, pielou evenness—J' and Shannon-Wiener, *H’* (log*e*)

**Table 3:** Distribution of culture-dependent *Trichoderma* spp., from coastal wetlands in Southeast China.

Species	Number of *Trichoderma* isolates										Total number
	Fengxian	Shanghai	Zhuhai	Shantou	Beihai	Fuzhou	Hangzhou	Ningbo	Lianyugang	Yantai	
*T*. *aureoviride*	0	3	1	0	0	0	2	6	0	0	12
*T*. *asperellum*	0	10	6	2	0	5	8	0	0	1	32
*T*. *harzianum*	1	5	12	3	0	4	8	29	0	1	63
*T*. *atroviride*	0	26	7	8	3	7	2	72	8	1	134
*T*. *koningiopsis*	0	0	0	0	0	0	1	0	0	1	2
*T*. *longibrachiatum*	0	1	0	1	0	0	2	0	0	0	4
*T*. *koningii*	0	0	0	0	0	0	1	0	0	0	1
*T*.*tawa*	0	0	0	0	0	0	1	0	0	0	1
*T*. *viridescens*,	0	0	0	0	0	0	1	0	0	0	1
*T*. *virens*	0	0	0	0	0	0	1	0	0	0	1
*T*.*hamatum*	0	0	0	0	0	0	0	1	0	0	1
*T*. *viride*	0	0	0	0	0	1	0	0	0	0	1
*T*. *velutinum*	0	0	0	1	0	0	0	0	0	0	1

### Phylogenetic analysis

The result of phylogenetic analysis based on the internal transcribed spacer regions 1 and 2 (ITS1 and ITS2) sequences of 52 *Trichoderma* isolates is shown in S1 Fig. The phylogenetic relationship of selected nucleotide sequences was inferred using the Maximum Parsimony method, and evolutionary analyses were conducted in MEGA6 [42]. The phylogenetic relationships of ITS 1, ITS 2 revealed that the *Viride* Clade of *T*. *asperellum* was distinguished in a single supported node with bootstrap support of 82%; *Green* Clade with *T*. *aureoviride; Green/Harzianum* Clade with *T*. *harzianum*, *T*. *tawa* and *T*. *velutinum* formed a distinguished single branch with the bootstrap support of 90–95%*; Longibrachiatum* Clade with *T*. *longibrachiatum* formed a separate group and showed the 70% bootstrap similarly with other Clades; *Virens* Clade with *T*. *virens* formed a separate group and showed the 83% bootstrap, similarly with *Green*/*Harzianum* Clade; *Viride* Clade with *T*. *atroviride*, *T*. *koningiopsis*, *T*. *koningii* and *T*. *viride* formed an individual group with the bootstrap support of 100% except for *T*. *koningii* which showed the 97% of similarly with its Clade of *Viride*. However, some strains were misplaced with distinguished Clade group (e.g *T*. *viride*, *T*. *longibrachiatum*, *T*. *atroviride*) because in few case internal transcribed spacer regions 1 and 2 (ITS1 and ITS2) could show the ambiguous identification of species [3, 7, 46].

In the case of unambiguous identification of *Trichoderma* isolates by ITS1 and ITS2, the fragment of the *tef* gene was sequenced and phylogenetic analysis was performed (S2 Fig). As a result of this analysis, *T*. *aureoviride*, *T*. *virens T*. *koningii*, *T*. *viride*, *T*. *atroviride*, *T*. *viridescens* were resolved with high bootstrap support. Sixty-three strains were identified as *T*. *harzianum*, but this species known to include several ITS alleles [46]. In addition the *tef1* tree clearly indicates the species complex of *T*. *harzianum* with the bootstrap support of only 17% with haplotypes and considered to be a species complex [32].

### Effect of sediment depth, sampling seasons, and sampling sites on culture-dependent *Trichoderma* biodiversity

*Trichoderma* counts were significantly influenced by sediment depth, sampling seasons, and sampling sites (S3 Table). *Trichoderma* counts were higher in the Ningbo, Zhejiang province and lower in Fengxian, Shanghai. It was 0.480±0.03 CFU.g^-1^ sediment in Ningbo and 0.100±0.05 CFU.g^-1^ sediment in Fengxian. *Trichoderma* counts varied from 0.004±0.01 to 0.685±0.03 CFU.g^-1^ sediment at different sediment depths. The count was maximum at 0–20 cm depth and minimum in 80–100 cm depth at two seasons. *Trichoderma* strain counts ranged from 0.17±0.02 to 0.26±0.02 CFU.g^-1^ sediment at two sampling stations. The count was maximum in summer and minimum in spring (S3 Table).

### Effect of sediment properties on culture-dependent *Trichoderma* biodiversity

*Trichoderma* counts were influenced by physical and chemical properties of sediment and the results are shown in the S4 Table. *Trichoderma* counts exhibited a positive correlation with temperature, Eh, and pH. All the correlated parameters were significant between sampling sites or seasons or sediment depths (S4 Table & S3 Fig). The significant correlation of pH revealed that while the pH was less than 5 (pH <5) the colonization of *Trichoderma* was reduced. Sediment temperature also played a significant role in the colonization of *Trichoderma* in sediment. Redox potential is a measure of oxygen in the sediment which revealed that sediment oxygen could enhance *Trichoderma* colonization.

Sediment temperature ranged from 15.57±0.32 to 25.54±0.21°C at different stations and it was higher in the Ningbo and minimum in the Chongming Island. In case of sediment depth it varied from 21.22±1.5 to 22.96±0.21°C at depths and it was maximum at 0–20 cm depth and minimum in 80–100 cm depth. In case of seasons, it ranged between 17.93±0.13 and 20.95±0.14°C at different seasons and it was maximum in summer and minimum in spring (S3 Table).

Redox potential ranged from -37.47±8.3 to -154.06±14.4 mV at different sites and it was higher in the Zhuhai and lower in the Chongming Island. In case of sediment depths, it ranged from -1.06±9.4 to 44.27±9.3 mV at depths and it was high at 0–20 cm depth and low at 40–60 cm depth. In case of seasons, it ranged between -66.93±5.89 and -89.36±5.94 mV at different seasons and it was maximum in summer and minimum in spring (S3 Table).

The pH was ranged from 7.55±0.12 to 8.61±0.1 at different sites and it was higher in the Fengxian and lower in the Shantou. In case of sediment depths, it ranged from 7.80±0.07 to 8.37±0.07 at depths and it was higher at 0–20 cm depth and lower in 80–100 cm depth. In case of seasons, it was ranged from 7.95±0.5 and 8.18±0.5 at different seasons and it was maximum in spring and minimum in summer (S3 Table).

### Biodiversity of culture independent *Trichoderma*

**Fig 1:**
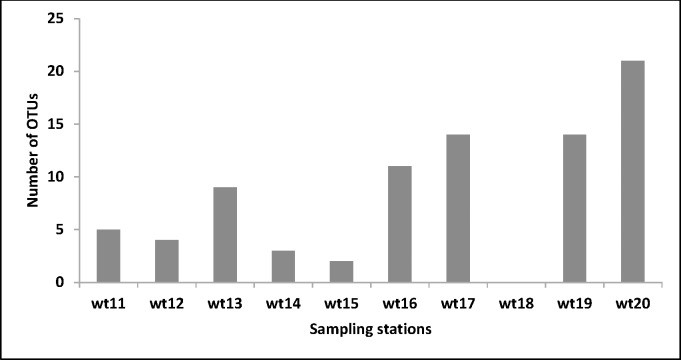
The number of operational taxanomical units (OTUs) found in different samplescollected from Southeast China [Zhuhai (wt11), Hangzhou (wt12), Ningbo (wt13), Fengxian (wt14), Yantai (wt15), Shanghai (wt16), Shantou (wt17), Beihai (wt18), Fuzhou (wt19) and Lianyungang (wt20)].

**Fig 2:**
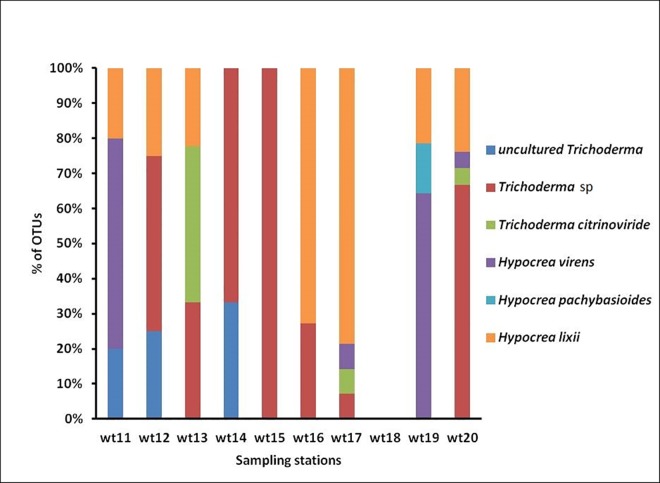
The percentage of *Trichoderma* spp., found in different sampling sites in Southeast China [Zhuhai (wt11), Hangzhou (wt12), Ningbo (wt13), Fengxian (wt14), Yantai (wt15), Shanghai (wt16), Shantou (wt17), Beihai (wt18), Fuzhou (wt19) and Lianyungang (wt20)].

**Fig 3:**
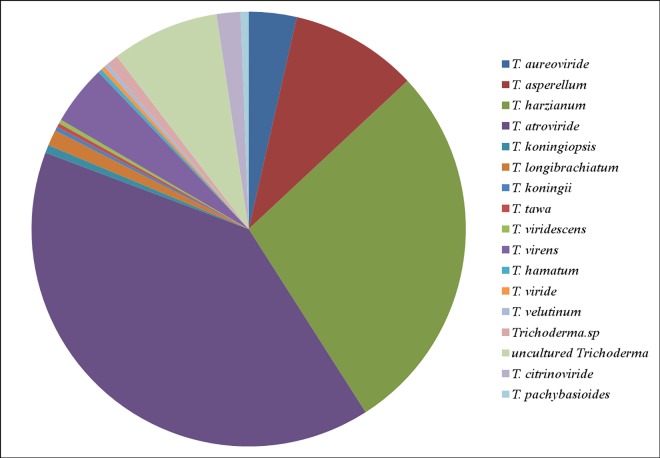
Biodiversity of culture-dependent and independent *Trichoderma* spp., from wetlands in Southeast China.

This work assessed the biodiversity of culture independent *Trichoderma* spp., from the selected representative sediment samples such as Zhuhai (wt11), Hangzhou (wt12), Ningbo (wt13), Fengxian (wt14), Yantai (wt15), Shanghai (wt16), Shantou (wt17), Beihai (wt18), Fuzhou (wt19) and Lianyungang (wt20). These samples were analyzed using the culture independent 454 pyrosequencing technique. A total of 83 18s rDNA *Trichoderma* sequences was obtained and used for classification. The dominant length distribution was found as 568 bp and overall six *Trichoderma* spp., were reported from the ten selected samples. The numbers of *Trichoderma* spp., were found higher in wt20 (21) and lower in wt18 (0) (Fig 1). Among these OTUs, 27 were uncultured *Trichoderma* OTUs (35%), 3 were *Trichoderma* sp., OTUs (8%), 6 were *T*. *citrinoviride* OTUs (6%), 14 were *T*. *virens* OTUs (14%), 2 were *T*. *polysporum* OTUs (1%), and 31 were *H*. *lixii* OTUs (26%) (Fig 2). The total biodiversity of *Trichoderma* spp., from coastal wetlands in China was analyzed by pooling the culture-dependent and independent results together and shown in Fig 3. A total of 17 including 16 known and one uncultured *Trichoderma* species were recorded by culture-dependent and independent methods.

### Effect of sampling sites on the culture independent *Trichoderma* spp.

**Fig 4:**
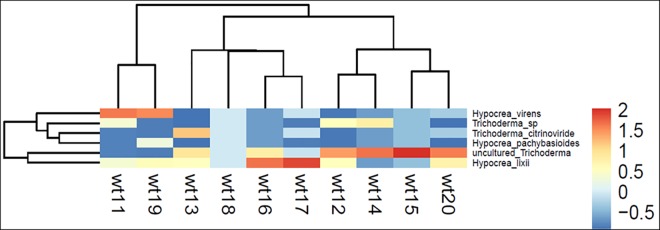
Heatmap analysis of OTUs of *Trichoderma* spp., based on the Bray- Curtis similarity of 454 pyroseqencing [Zhuhai (wt11), Hangzhou (wt12), Ningbo (wt13), Fengxian (wt14), Yantai (wt15), Shanghai (wt16), Shantou (wt17), Beihai (wt18), Fuzhou (wt19) and Lianyungang (wt20)].

The heatmap was drawn based on the *Trichoderma* spp., in accordance with the sampling sites, which were classified into the four groups (Fig 4). The abundance and richness of the uncultured *Trichoderma* spp., were higher in wt14 (33.33%), *Trichoderma* sp., was higher in wt15 (100%), *T*. *citrinoviride* was higher in wt12 (44.44%), *T*. *virens* was higher in wt11 (60%), *T*. *polysporum* was higher in wt18 (14.28%), and *H*. *lixii* was higher in wt15 (72.72%). Regarding species relationship the *H*. *lixii* and unculturable *Trichoderma* showed significant similarity and formed as one group (Fig 4).

### Effect of sediemnt properties on culture independent *Trichoderma* spp.

**Fig 5:**
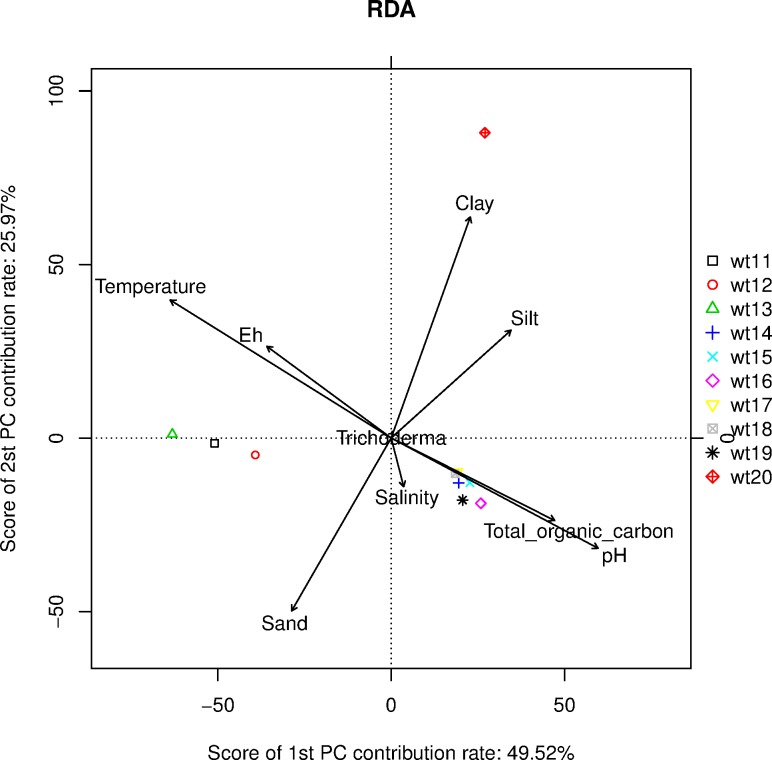
Redundancy discriminant analysis (RDA) biplot of the distributiion of *Trichoderma* spp., with sediment properties. [Zhuhai (wt11), Hangzhou (wt12), Ningbo (wt13), Fengxian (wt14), Yantai (wt15), Shanghai (wt16), Shantou (wt17), Beihai (wt18), Fuzhou (wt19) and Lianyungang (wt20)].

The effect of sediemnt properties on distribution of culture independent *Trichoderma* spp.,was determined by Redundancy Discriminant Analysis (RDA) (Fig 5; S5 Table) The eigenvalues of the sediment properties was explained for 1,142,651 with the significant explanatory power of the RDA. According to the species variation data, a score of 1^st^ PC contribution was 49.52% and the score of the 2^nd^ PC contribution rate was 25.97%. The soil properties such as temperature, Eh and pH were significant at 5% level *P* values based on 999 permutations. The RDA analysis revealed that all the sediment properties were moderately influenced the culture independent *Trichoderma* biodiversity. Satatstical analsyis of relation between the sediemnt properties and culture-dependent and independent *Trichoderma* counts revealed that the temperature and Eh were significant factors and highly influenced the *Trichoderma* biodiversity (Fig 5, S3 Fig & S6 Table).

## Discussion

Ninety-one *Trichoderma* spp., are previously been reported from China [7, 8]. However, the China coastal ecosystem, interface between terrestrial and marine biotopes are significant in extent in Southeast China but has not been studied the distribution and/or ecology of *Trichoderma* spp. Hence this work assessed the culture-dependent and independent biodiversity of *Trichoderma* spp., from coastal wetlands in Southeast China by traditional molecular (culture-dependent) and 454 Pyrosequencing (culture independent) methods.

Here and the previous study, *T*. *harzianum* was the predominant taxon [3, 20, 32, 46–49]. *Trichoderma harzianum* is frequently recorded in the genus, occurring in diverse ecosystem [19]. The species identified in the present study were: *T*. *koningiopsis* has been often isolated from natural substrata in tropical America, East Africa, Europe and Canada, and from ascospores [24]. *Trichoderma hamatum* isolated from soil are wildly used for the biocontrol of plant diseases caused by *Sclerotinia sclerotiorum* and *Rhizoctonia solani* and also trigger the plant growth [50,51]. *Trichoderma longibrachiatum* is isolated from *Cerastoderma edule* (Mollusc) [52] and *T*. *viride* is isolated from the rhizosphere soil of *Avicennia marina* with the nematocidal activity [53]. *Trichoderma aureviride* is isolated from the sea sediments of South China and showed the significant lytic enzyme activity [54] and *T*. *atroviride* is isolated from sediments of the root of mangroves *Ceriops tagal* and sponge *Psammocinia* sp. [55, 56]. *Trichoderma harzianum* is isolated from marine sponge *Gelliodes fibrosa* & *Suberites zeteki*, *Tethya aurantium* with the antimicrobial activity [56–60].

The present study reports 13 *Trichoderma* spp., including a new record of *T*. *tawa*, from Hangzhou. *Trichoderma* spp., were significantly diversified in Hangzhou (10 species) and Ningbo (4 species), both are in Zhejiang province is provided a high biodiversity of *Trichoderma* spp., due to the extensive ecological conditions found in the coastline of this province. Several researchers have reported that the plant's litter decomposition supported higher microbial colonization because of nutrient richness [61–63]. We found that *T*. *atroviride* (134 isolates) followed by *H*. *lixii* (63) were the most common species in our study sites this result was supported both by culture-dependent and independent methods. Especially, *H*. *lixii*/ *Trichoderma harzianum* is a significantly predominant species in China and this was also supported by 454 pyrosequencing analysis showing higher 26% OTUs of *H*. *lixii* compared with other reported species. This result is consistent with previous reports of *Trichoderma* biodiversity in China [3, 25, 45, 48].

The comparison of the culture-dependent and independent *Trichoderma* analysis revealed that the culture-dependent *Trichoderma* biodiversity was 46.15% higher than the culture independent *Trichoderma* biodiversity, as assessed by 454 pyrosequencing. The comparison of the pyrosequencing with traditional identification (CD) methods could enable us understating the *Trichoderma* biodiversity. *Trichoderma citrinoviride* and *T*. *polysporum* were found in 454 pyrosequencing but not in traditional (CD) methods, whereas *Trichoderma harzianum* and *T*. *virens* were commonly found in both traditional (CD) and 454 pyrosequencing methods. These results indicated the drawback in 454 pyrosequencing analysis based on ITS sequencing in estimating *Trichoderma* biodiversity [64].

Redundancy discriminant analysis (RDA) biplot and correlations analysis of the environmental variables revealed that the temperature, Eh and pH significantly influenced the biodiversity of culture-dependent and independent *Trichoderma* spp. The ecological variance is an important factor for the colonization of the microbes in the sediment [47, 64]. Temporal and ecological conditions of sediments could influence the distribution of the *Trichoderma* spp. [47, 65]. Hence, the climatic, spatial and temporal changes can play a significant role in the distribution of *Trichoderma* spp., in China.

## Conclusion

The present study assessed the biodiversity of the *Trichoderma* spp., by culture-dependent and independent methods. A total of 13 *Trichoderma* spp., including a new report of *T*. *tawa* was recovered by traditional method (culture-depentent). In contrast, culture independent pyrosequencing, we identified a total of six *Trichoderma* spp. Thus both methods could provide complementary advantages; in other words, the combination of these two methods could reveal more biodiversity of *Trichoderma* spp. Since 454 pyrosequencing is an advanced method to study the culture independent microbes, the proper designing of primers and availability of specific database is required according to the microbe’s specificity and it could enable the obvious study of microbial diversity. The sediment properties of temperature, Eh and pH could significantly influence the biodiversity of *Trichoderma* spp., from coastal wetlands in Southeast China.

## Supporting Information

S1 Fig Phylogenetic relationships of ITS 1, ITS 2 sequences obtained from 52 *Trichoderma* isolates inferred by parsimony analysis are listed in gene bank accession numbers in Table 1. The numbers given over branches indicates the bootstrap value (expressed as a percentage of 1,000 replicates) greater than 50% are at given branches.
(DOC)

S2 Fig Phylogenetic relationships of *tef-α* sequences obtained from 52 isolates inferred by parsimony analysis are listed in gene bank accession numbers Table 1. The numbers given over branches indicates the bootstrap coefficient value (expressed as a percentage of 1,000 replicates) greater than 50% are at given branches.
(DOC)

S3 Fig Relationships between culture-dependent *Trichoderma* counts and sediment properties (DOC)

S1 Table Study area description. (DOC)

S2 Table Total *Trichoderma* species reported in this study. (DOC)

S3 Table Physical and chemical characteristics and *Trichoderma* counts (CFU) of soil in sampling stations in five soil depths in two seasons. 6 univariate 2- way ANOVAs used followed by multiple comparison teats (SNK and Tukey’s) to examine effects of genotype species and season on seven biochemical response variables. ** = p < 0.01;* = p < 0.05; NS = Not Significant.
(DOC)

S4 Table *Trichoderma* counts in relation to varied physical- chemical factors. (DOC)

S5 Table *Trichoderma* species richness index. (DOC)

S6 Table Redundancy discriminant analysis (RDA) of the distribution of *Trichoderma* species with environmental variables of the study stations significant codes: '*' 0.05 ' ‘NS’ not significant. P values based on 999 permutations.
(DOC)
