# Supplementary material for: Biodiversity of Trichoderma Community in the Tidal Flats and Wetland of Southeastern China
Source: PLoS One. 2016 Dec 21;11(12):e0168020. doi: 10.1371/journal.pone.0168020 (PMC5176281; doi:10.1371/journal.pone.0168020)

S1 Fig Phylogenetic relationships of ITS 1, ITS 2 sequences obtained from 52 *Trichoderma* isolates inferred by parsimony analysis are listed in gene bank accession numbers Table 1. The numbers given over branches indicates the bootstrap value (expressed as a percentage of 1,000 replicates) greater than 50 % are at given branches.


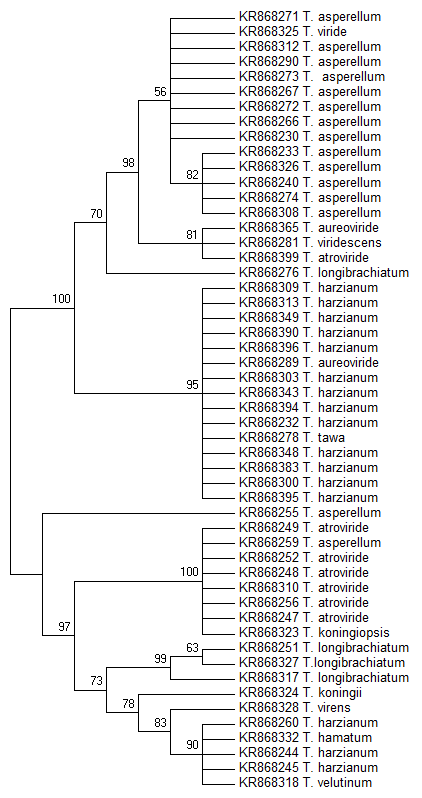

Supplement: S1 Fig — The numbers given over branches indicates the bootstrap value (expressed as a percentage of 1,000 replicates) greater than 50% are at given branches. (DOC) [file pone.0168020.s001.doc]
