# Supplementary figures and images for: Biodiversity of Trichoderma Community in the Tidal Flats and Wetland of Southeastern China
Source: PLoS One. 2016 Dec 21;11(12):e0168020. doi: 10.1371/journal.pone.0168020 (PMC5176281; doi:10.1371/journal.pone.0168020)

S3 Fig Relationships between culture-dependent *Trichoderma* counts and sediment properties


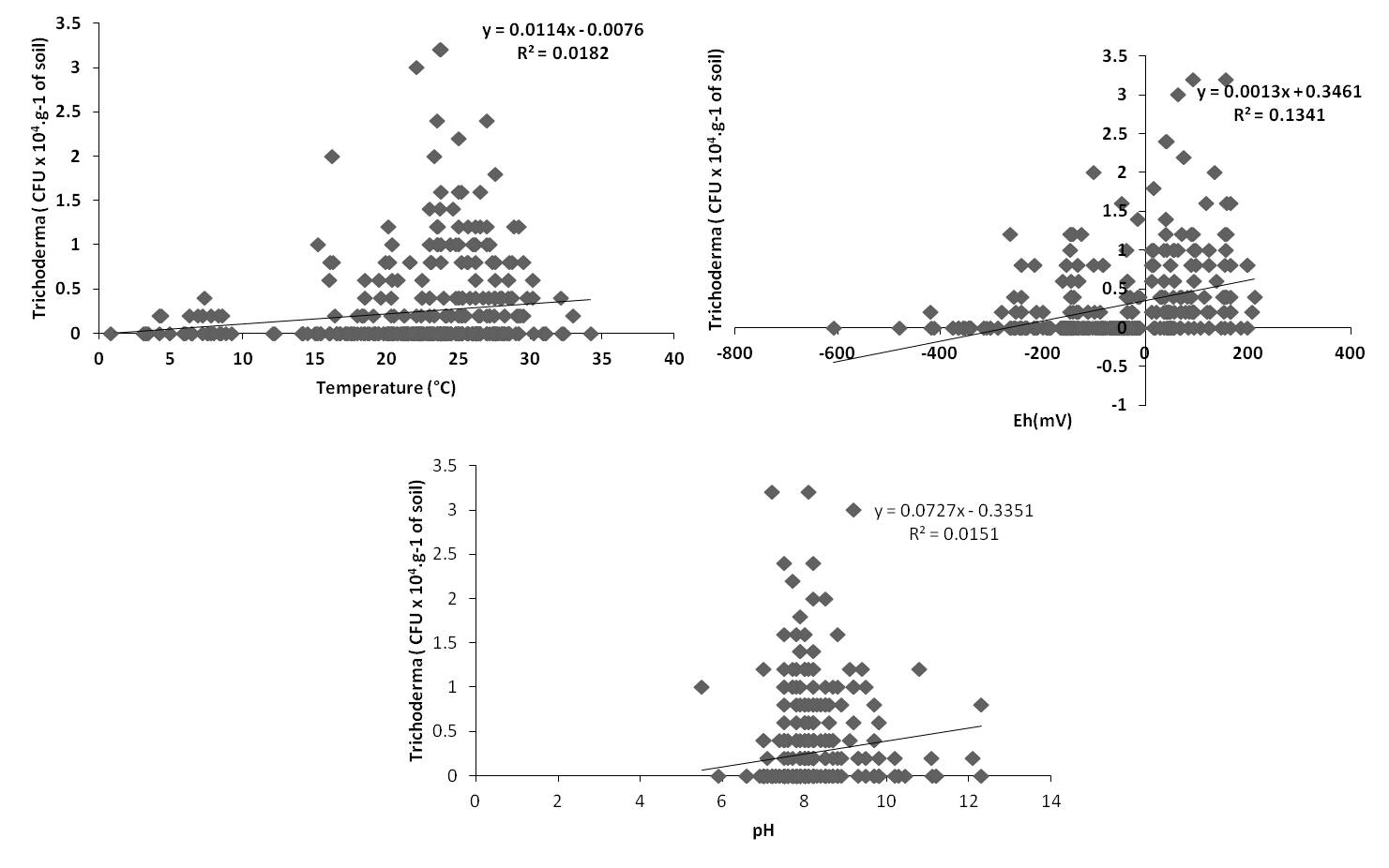

Supplement: S3 Fig — (DOC) [file pone.0168020.s003.doc]
