# Supplementary material for: Biodiversity of Trichoderma Community in the Tidal Flats and Wetland of Southeastern China
Source: PLoS One. 2016 Dec 21;11(12):e0168020. doi: 10.1371/journal.pone.0168020 (PMC5176281; doi:10.1371/journal.pone.0168020)
Supplement: S1 Table — (DOC) [file pone.0168020.s004.doc]

**S1 Table** Study area description

| **PROVINCE** | **STATIONS** | **TRANSECTS** | **LOT. LONG.** |
| --- | --- | --- | --- |
| **Guangxi** | **Behai (Mangroves wetland)** | *Avicennia marina* zone | 21°25’11.49”N;109°12’ 53.54”E |
|  |  | *Kandida candle* zone | 21°25’12.68”N; 109°13’ 01.04”E |
|  |  | *Luxuriant Avicennia marina* zone | 21°25’13.27”N; 109°13’ 01.93”E |
|  |  | *Avicennia alba* zone | 21°25’12.41”N; 109°13’ 08.98”E |
| **Guangdong** | **Zhuhai** | Yinsha beach | 22°00’20.80”N; 113°20’ 25.75”E |
|  |  | Aquaculture system, Yinsha beach | 22°01’46.01”N; 113°23’ 26.39”E |
|  |  | Mangroves wetland park | 22°25’42.65”N; 113°37’ 51.75”E |
|  |  | *Bruguiera gymnorrhiza zone* | 22°25’38.81”N; 113°37’ 38.75”E |
|  |  | *Bruguiera sexangula zone* | 22°25’40.49”N; 113°37’ 37.54”E |
|  |  | Aquaculture system | 22°25’42.22”N; 113°37’ 52.89”E |
|  |  | *Excoecaria agallocha zone* | 22°25’38.53”N; 113°37’ 39.15”E |
|  |  | *Rhizophora apiculata zone* | 22°25’36.70”N; 113°37’ 43.59”E |
|  |  |  |  |
|  | **Shantou** | Wetland ecosystem | 23°21’35.49”N; 116°39’ 38.29”E |
|  |  | Aquaculture system | 23°26’09.78”N; 117°00’ 37.67”E |
|  |  | Wetland | 23°26’54.95”N; 117°00’ 06.01”E |
| **Fujian** | **Fuzhou** | Wetland beach | 26°03’57.25”N; 119°37’ 42.36”E |
|  |  | Wetland soil | 26°03’57.30”N; 119°37’ 45.72”E |
|  |  | River cannel | 26°03’55.26”N; 119°36’ 02.79”E |
|  |  |  |  |
| **Shanghai** | **Chongming** | Yangzi river Chumining island (Dongtan wetland park | 31°32'33.13"N;121°50'37.93"E |
|  |  | Bihai Jinsha Beach park | 30°49'16.18"N;121°32'46.61"E |
|  |  | Xixa park west sand wetland | 31°43'31.76"N;121°14'01.71"E |
|  |  | Huangpu river cannel (Binjiang forest park) | 31°23'20.20"N;121°31'13.89"E |
|  |  |  |  |
|  | **Fengxian** | Huangpu River(sand factory) | 31°01'55.88"N; 21°28'35.28"E |
|  |  | huangpu River(Pujiang base) | 31°02'44.91"N; 21°28'52.29"E |
|  |  | Hangpu & yangci River eastuary | 31°23'20.75"N;121°31'10.16"E |
|  |  |  |  |
| **Zhejiang** | **Hangzhou** | Wetland park | 30°16'31.42"N;120°04'07.52"E |
|  |  | Botanical garden wetland | 30°16'32.32"N;120°04'20.93"E |
|  |  | Underwater ecology observation corridor | 30°16'27.03"N;120°04'17.09"E |
|  |  | Qian-tang river North | 30°16'14.04"N;120°20'16.48"E |
|  |  | Qian-tang river South | 30°17'37.63"N;120°16'04.12"E |
|  | **Nigbo** | Beach | 29°15'13.43"N;121°57'35.65"E |
|  |  | Coastal form | 29°15'03.18"N;121°57'18.84"E |
|  |  | Aquaculture form | 29°15'00.65"N;121°56'45.78"E |
|  |  | Horbur | 29°12'11.80"N;121°56'56.27"E |
|  |  | Wetland park | 29°12'55.69"N;121°57'39.97"E |
|  |  | Reservoir | 29°13'06.30"N;121°56'11.92"E |
|  |  | Estuary | 29°14'03.90"N;121°57'08.30"E |
| **Jiangsu** | **Lianyugang** | Beach 1 | 34°45'29.54"N;119°22'02.97"E |
|  |  | Beach 2 | 34°41'19.41"N;119°16'32.54"E |
|  |  | Lianyugang Island soil | 34°45'44.78"N;119°28'22.30"E |
|  |  | Aquaculture form system | 34°45'29.78"N;119°12'01.44"E |
|  |  | River soil | 34°45'25.75"N;119°12'10.71"E |
