# Supplementary material for: Biodiversity of Trichoderma Community in the Tidal Flats and Wetland of Southeastern China
Source: PLoS One. 2016 Dec 21;11(12):e0168020. doi: 10.1371/journal.pone.0168020 (PMC5176281; doi:10.1371/journal.pone.0168020)
Supplement: S2 Table — (DOC) [file pone.0168020.s005.doc]

**S2 Table Total *Trichoderma* species reported in this study**

| **Isolation source** |  | **Culture collection** | **Strain** | **Organism** | **Clade** |
| --- | --- | --- | --- | --- | --- |
|  |  |  |  |  |  |
| Sediment, Hangzhou wetland park, Botanical garden | Hangzhou | CCTCC-RW0004 | ZWPBG1 | *Trichoderma aureoviride* | Green |
| Soil,wetland forest, Chongming, Shanghai | Shanghai | CCTCC-SBW0007 | CHI1 | *Trichoderma asperellum* | Viride |
| Rhizosphere soil, Mangroves-Rhizophora apiculata, Zhuhai | Zhuhai | CCTCC-SBW0102 | ZHMT8 | *Trichoderma asperellum* | Viride |
| Rhizosphere soil, Mangroves-Avicennia alba zone Zhuhai | Zhuhai | CCTCC-SBW0108 | ZHMT9 | *Trichoderma harzianum* | Green/Harzianum |
| Rhizosphere soil, Mangroves- Avicennia marina zone, Zhuhai | Zhuhai | CCTCC-SBW0106 | ZHMT7 | *Trichoderma asperellum* | Viride |
| Rhizosphere soil, Mangroves-Avicennia alba zone, Zhuhai | Zhuhai | CCTCC-SBW0098 | ZHMT2 | *Trichoderma harzianum* | Green/Harzianum |
| Rhizosphere soil, Mangroves-Rhizophora apiculata, Jinsha park, Zhuhai | Zhuhai | CCTCC-SBW0005 | BJ1 | *Trichoderma aureoviride* | Green |
| Rhizosphere soil, Mangroves-Avicennia marina zone Zhuhai | Zhuhai | CCTCC-SBW0095 | ZHMT12 | *Trichoderma harzianum* | Green/Harzianum |
| Rhizosphere soil, Mangroves-Rhizophora apiculata, Zhuhai | Zhuhai | CCTCC-SBW0101 | ZHMT3 | *Trichoderma harzianum* | Green/Harzianum |
| Rhizosphere soil, Mangroves-Avicennia marina zone, Zhuhai | Zhuhai | CCTCC-SBW0109 | ZHMT9 | *Trichoderma asperellum* | Viride |
| Rhizosphere soil, Mangroves-Bruguiera gymnorrhiza, Zhuhai | Zhuhai | CCTCC-SBW0103 | ZHMT5 | *Trichoderma harzianum* | Green/Harzianum |
| Rhizosphere soil, Mangroves-Bruguiera gymnorrhiza, Zhuhai | Zhuhai | CCTCC-SBW0105 | ZHMT6 | *Trichoderma asperellum* | Viride |
| Rhizosphere soil, Mangroves-Avicennia marina zone, Zhuhai | Zhuhai | CCTCC-SBW0094 | ZHMT1 | *Trichoderma harzianum* | Green/Harzianum |
| Soil, Fengxian | Fengxian | CCTCC-RW0001 | CHIPU1 | *Trichoderma harzianum* | Green/Harzianum |
| Rhizosphere soil, Mangroves-Avicennia marina zone Zhuhai | Zhuhai | CCTCC-SBW0096 | ZHMT13 | *Trichoderma harzianum* | Green/Harzianum |
| Soil, Yinsha beach, Zhuhai | Zhuhai | CCTCC-SBW0114 | ZHYAQT2 | *Trichoderma harzianum* | Green/Harzianum |
| Rhizosphere soil, Mangroves-Avicennia marina zone, Zhuhai | Zhuhai | CCTCC-SBW0097 | ZHMT14 | *Trichoderma harzianum* | Green/Harzianum |
| Soil, Wetland east forest Chongming, Shanghai | Shanghai | CCTCC-SBW0015 | CHI5 | *Trichoderma asperellum* | Viride |
| Soil, Wetland east forest, Chongming, Shanghai | Shanghai | CCTCC-SBW0022 | CHIPUFI2 | *Trichoderma atroviride* | Viride |
| Soil, Wetland forest, Chongming, Shanghai | Shanghai | CCTCC-SBW0021 | CHIA | *Trichoderma atroviride* | Viride |
| Soil, Wetland south forest, Chongming, Shanghai | Shanghai | CCTCC-SBW0016 | CHI5 | *Trichoderma atroviride* | Viride |
| Soil, Wetland forest, Chongming, Shanghai | Shanghai | CCTCC-SBW0009 | CHI12 | *Trichoderma atroviride* | Viride |
| Soil, Wetland forest, Chongming, Shanghai | Shanghai | CCTCC-SBW0023 | PU2 | *Trichoderma longibrachiatum* | Longibrachiatum |
| Soil, Wetland north forest, Chongming, Shanghai | Shanghai | CCTCC-SBW0008 | CHI11 | *Trichoderma atroviride* | Viride |
| Soil, Wetland west forest, Chongming, Shanghai | Shanghai | CCTCC-SBW0025 | PUFP3 | *Trichoderma atroviride* | Viride |
| Soil, Wetland forest, Chongming, Shanghai | Shanghai | CCTCC-SBW0017 | CHI6 | *Trichoderma asperellum* | Viride |
| Soil, Wetland forest, Chongming, Shanghai | Shanghai | CCTCC-SBW0011 | CHI2 | *Trichoderma asperellum* | Viride |
| Soil, Wetland forest, Chongming, Shanghai | Shanghai | CCTCC-SBW0024 | PUFP1 | *Trichoderma atroviride* | Viride |
| Soil, Wetland south forest, Chongming, Shanghai | Shanghai | CCTCC-SBW0014 | CHI4 | *Trichoderma asperellum* | Viride |
| Soil, Wetland forest, Chongming, Shanghai | Shanghai | CCTCC-SBW0012 | CHI3 | *Trichoderma asperellum* | Viride |
| Soil, Wetland forest, Chongming, Shanghai | Shanghai | CCTCC-SBW0019 | CHI8 | *Trichoderma asperellum* | Viride |
| Rhizosphere soil, Mangroves-Avicennia marina zone Zhuhai | Zhuhai | CCTCC-SBW0100 | ZHMT21 | *Trichoderma harzianum* | Green/Harzianum |
| Soil, Wetland forest, Chongming, Shanghai | Shanghai | CCTCC-SBW0013 | CHI4 | *Trichoderma asperellum* | Viride |
| Soil, Wetland forest, Chongming, Shanghai | Shanghai | CCTCC-SBW0010 | CHI15 | *Trichoderma asperellum* | Viride |
| Soil, wetland, Fuzhou | Fuzhou | CCTCC-SBW0057 | FJWT7 | *Trichoderma asperellum* | Viride |
| Water, Shantou river | Shantou | CCTCC-SBW0089 | SYC2 | *Trichoderma atroviride* | Viride |
| Soil, wetland, Fuzhou | Fuzhou | CCTCC-SBW0050 | FJWT1 | *Trichoderma harzianum* | Green/Harzianum |
| Soil, wetland, Fuzhou | Fuzhou | CCTCC-SBW0054 | FJWT4 | *Trichoderma asperellum* | Viride |
| Soil, wetland, Fuzhou | Fuzhou | CCTCC-SBW0052 | FJWT2 | *Trichoderma asperellum* | Viride |
| Soil, Yantai, Shandong | Yantai | CCTCC-SBW0092 | SYXC3 | *Trichoderma atroviride* | Viride |
| Soil, wetland, Fuzhou | Fuzhou | CCTCC-SBW0055 | FJWT5 | *Trichoderma harzianum* | Green/Harzianum |
| Soil, Yantai, Shandong | Yantai | CCTCC-SBW0090 | STWT5 | *Trichoderma koningiopsis* | Viride |
| Soil, wetland, Fuzhou | Fuzhou | CCTCC-SBW0058 | FJWT8 | *Trichoderma asperellum* | Viride |
| Soil, wetland, Fuzhou | Fuzhou | CCTCC-SBW0053 | FJWT3 | *Trichoderma asperellum* | Viride |
| Sediment, Wetland, Shantou | Shantou | CCTCC-SBW0080 | STWT4 | *Trichoderma asperellum* | Viride |
| Sediment, Wetland , Shantou | Shantou | CCTCC-SBW0084 | STWT6 | *Trichoderma asperellum* | Viride |
| Sediment, wetland park, Hangzhou | Hangzhou | CCTCC-RW0021 | ZWPUEB12 | *Trichoderma harzianum* | Green/Harzianum |
| Soil Qian Tang River, Hangzhou | Hangzhou | CCTCC-RW0002 | ZQTR1 | *Trichoderma longibrachiatum* | Longibrachiatum |
| Sediment, Botanical garden, wetland park, Hangzhou | Hangzhou | CCTCC-RW0008 | ZWPBG3 | *Trichoderma atroviride* | Viride |
| Sediment, wetland park, Hangzhou | Hangzhou | CCTCC-RW0023 | ZWPUEB14 | *Trichoderma tawa* | Green/Harzianum |
| Sediment, wetland park, Hangzhou | Hangzhou | CCTCC-RW0022 | ZWPUEB13 | *Trichoderma harzianum* | Green/Harzianum |
| Sediment, wetland park, Hangzhou | Hangzhou | CCTCC-RW0019 | ZWPUEB10 | *Trichoderma harzianum* | Green/Harzianum |
| Sediment, wetland park, Hangzhou | Hangzhou | CCTCC-RW0017 | ZWPH1 | *Trichoderma viridescens* | Viride |
| Sediment, Botanical garden, wetland park, Hangzhou | Hangzhou | CCTCC-RW0011 | ZWPBG6 | *Trichoderma asperellum* | Viride |
| Sediment, wetland park, Hangzhou | Hangzhou | CCTCC-RW0024 | ZWPUEB15 | *Trichoderma harzianum* | Green/Harzianum |
| Sediment, Botanical garden, wetland park, Hangzhou | Hangzhou | CCTCC-RW0006 | ZWPBG10 | *Trichoderma harzianum* | Green/Harzianum |
| Sediment, Botanical garden, wetland park, Hangzhou | Hangzhou | CCTCC-RW0013 | ZWPBG8 | *Trichoderma asperellum* | Viride |
| Sediment, Botanical garden, wetland park, Hangzhou | Hangzhou | CCTCC-RW0005 | ZWPBG1 | *Trichoderma asperellum* | Viride |
| Sediment, Botanical garden, wetland park, Hangzhou | Hangzhou | CCTCC-RW0014 | ZWPBG8 | *Trichoderma asperellum* | Viride |
| Sediment, Botanical garden, wetland park, Hangzhou | Hangzhou | CCTCC-RW0015 | ZWPBG9 | *Trichoderma harzianum* | Green/Harzianum |
| Sediment, wetland park, Hangzhou | Hangzhou | CCTCC-RW0003 | ZEPUEB7 | *Trichoderma aureoviride* | Green |
| Sediment, Botanical garden, wetland park, Hangzhou | Hangzhou | CCTCC-RW0007 | ZWPBG2 | *Trichoderma asperellum* | Viride |
| Sediment, Botanical garden, wetland park, Hangzhou | Hangzhou | CCTCC-RW0026 | ZWPUEB2 | *Trichoderma atroviride* | Viride |
| Estuary soil, Ningbo, Zhejiang | Ningbo | CCTCC-SBW0184 | ZNE8 | *Trichoderma harzianum* | Green/Harzianum |
| Estuary soil, Ningbo, Zhejiang | Ningbo | CCTCC-SBW0185 | ZNE9 | *Trichoderma harzianum* | Green/Harzianum |
| Estuary soil, Ningbo, Zhejiang | Ningbo | CCTCC-SBW0181 | ZNE5 | *Trichoderma harzianum* | Green/Harzianum |
| Estuary soil, Ningbo, Zhejiang | Ningbo | CCTCC-SBW0179 | ZNE1 | *Trichoderma harzianum* | Green/Harzianum |
| Beach water, Ningbo, Zhejiang | Ningbo | CCTCC-SBW0156 | ZNBW12 | *Trichoderma harzianum* | Green/Harzianum |
| Beach water, Ningbo, Zhejiang | Ningbo | CCTCC-SBW0154 | ZNBW10 | *Trichoderma harzianum* | Green/Harzianum |
| Coastal Form water, Ningbo, Zhejiang | Ningbo | CCTCC-SBW0178 | ZNCFW8 | *Trichoderma atroviride* | Viride |
| Aquaculture form water, Ningbo, Zhejiang | Ningbo | CCTCC-SBW0129 | ZNAF15 | *Trichoderma atroviride* | Viride |
| Coastal form soil, Ningbo, Zhejiang | Ningbo | CCTCC-SBW0169 | ZNCF17 | *Trichoderma harzianum* | Green/Harzianum |
| Coastal Form water, Ningbo, Zhejiang | Ningbo | CCTCC-SBW0177 | ZNCFW7 | *Trichoderma atroviride* | Viride |
| Coastal form soil, Ningbo, Zhejiang | Ningbo | CCTCC-SBW0170 | ZNCF18 | *Trichoderma atroviride* | Viride |
| Beach water, Ningbo, Zhejiang | Ningbo | CCTCC-SBW0155 | ZNBW11 | *Trichoderma harzianum* | Green/Harzianum |
| Beach water, Ningbo, Zhejiang | Ningbo | CCTCC-SBW0158 | ZNBW15 | *Trichoderma harzianum* | Green/Harzianum |
| Reservoir soil, Ningbo, Zhejiang | Ningbo | CCTCC-SBW0205 | ZNR19 | *Trichoderma atroviride* | Viride |
| Reservoir soil, Ningbo, Zhejiang | Ningbo | CCTCC-SBW0208 | ZNR4 | *Trichoderma harzianum* | Green/Harzianum |
| Mangrove rhizosphere soil, Beihai | Ningbo | CCTCC-SBW0004 | BHMT4 | *Trichoderma harzianum* | Green/Harzianum |
| Mangroves rhizosphere soil, Zhuhai | Zhuhai | CCTCC-SBW0102 | ZHMT4 | *Trichoderma asperellum* | Viride |
| Soil, wetland forest, Chongming, Shanghai | Shanghai | CCTCC-SBW0006 | CHI (WIN) | *Trichoderma harzianum* | Green/Harzianum |
| Soil, wetland forest, Chongming, Shanghai | Shanghai | CCTCC-SBW0018 | CHI7 | *Trichoderma atroviride* | Viride |
| Soil, wetland forest, Chongming, Shanghai | Shanghai | CCTCC-SBW0020 | CHI9 | *Trichoderma asperellum* | Viride |
| Mangroves rhizosphere soil, Zhuhai | Zhuhai | CCTCC-SBW0104 | ZHMT5 | *Trichoderma asperellum* | Viride |
| Mangroves rhizosphere soil, Zhuhai | Zhuhai | CCTCC-SBW0099 | ZHMT20 | *Trichoderma harzianum* | Green/Harzianum |
| Soil, Yantai, Shandong | Yantai | CCTCC-SBW0091 | SYXC1 | *Trichoderma asperellum* | Viride |
| Soil, Yantai, Shandong | Yantai | CCTCC-SBW0093 | SYXR1 | *Trichoderma harzianum* | Green/Harzianum |
| Soil, wetland, Fuzhou | Fuzhou | CCTCC-SBW0056 | FJWT6 | *Trichoderma atroviride* | Viride |
| Wetland sediment, Shantou | Shantou | CCTCC-SBW0076 | STWT1 | *Trichoderma longibrachiatum* | Longibrachiatum |
| water sample, wetland, Shantou | Shantou | CCTCC-SBW0088 | SW2 | *Trichoderma velutinum* | Green/Harzianum |
| Sediment, wetland park, Hangzhou | Hangzhou | CCTCC-RW0027 | ZWPUEB3 | *Trichoderma asperellum* | Viride |
| Sediment, wetland park, Hangzhou | Hangzhou | CCTCC-RW0018 | ZWPUEB | *Trichoderma harzianum* | Green/Harzianum |
| Sediment, wetland park, Hangzhou | Hangzhou | CCTCC-RW0020 | ZWPUEB11 | *Trichoderma harzianum* | Green/Harzianum |
| Sediment, Botanical garden, wetland park, Hangzhou | Hangzhou | CCTCC-RW0012 | ZWPBG7 | *Trichoderma asperellum* | Viride |
| Sediment, Botanical garden, wetland park, Hangzhou | Hangzhou | CCTCC-RW0009 | ZWPBG4 | *Trichoderma koningiopsis* | Viride |
| Sediment, Botanical garden, wetland park, Hangzhou | Hangzhou | CCTCC-RW0010 | ZWPBG5 | *Trichoderma koningii* | Viride |
| Wetland soil, Fuzhou | Fuzhou | CCTCC-BW0051 | FJWT14 | *Trichoderma viride* | Viride |
| Sediment, wetland park, Hangzhou | Hangzhou | CCTCC-RW0028 | ZWPUEB4 | *Trichoderma asperellum* | Viride |
| Sediment, wetland park, Hangzhou | Hangzhou | CCTCC-RW0025 | ZWPUEB2 | *Trichoderma longibrachiatum* | Longibrachiatum |
| Sediment, Botanical garden, wetland park, Hangzhou | Hangzhou | CCTCC-RW0016 | ZWPBG9 | *Trichoderma virens* | Green |
| Estuary soil, Ningbo, Zhejiang | Ningbo | CCTCC-SBW0183 | ZNE7 | *Trichoderma harzianum* | Green/Harzianum |
| Wetland soil, Ningbo, Zhejiang | Ningbo | CCTCC-SBW0224 | ZNWPL8 | *Trichoderma atroviride* | Viride |
| Coastal form soil, Ningbo, Zhejiang | Ningbo | CCTCC-SBW0171 | ZNCF20 | *Trichoderma atroviride* | Viride |
| Aquaculture form soil, Ningbo, Zhejiang | Ningbo | CCTCC-SBW0136 | ZNAF20 | *Trichoderma hamatum* | Viride |
| Aquaculture form soil, Ningbo, Zhejiang | Ningbo | CCTCC-SBW0139 | ZNAF22 | *Trichoderma atroviride* | Viride |
| Harbor soil, Ningbo, Zhejiang | Ningbo | CCTCC-SBW0188 | ZNH11 | *Trichoderma atroviride* | Viride |
| Harbor soil, Ningbo, Zhejiang | Ningbo | CCTCC-SBW0190 | ZNH12 | *Trichoderma atroviride* | Viride |
| Harbor soil, Ningbo, Zhejiang | Ningbo | CCTCC-SBW0192 | ZNH14 | *Trichoderma harzianum* | Green/Harzianum |
| Harbor soil, Ningbo, Zhejiang | Ningbo | CCTCC-SBW0191 | ZNH13 | *Trichoderma atroviride* | Viride |
| Aquaculture form soil, Ningbo, Zhejiang | Ningbo | CCTCC-SBW0137 | ZNAF21 | *Trichoderma atroviride* | Viride |
| Aquaculture form soil, Ningbo, Zhejiang | Ningbo | CCTCC-SBW0144 | ZNAF29 | *Trichoderma atroviride* | Viride |
| Aquaculture form soil, Ningbo, Zhejiang | Ningbo | CCTCC-SBW0145 | ZNAF30 | *Trichoderma atroviride* | Viride |
| Harbor soil, Ningbo, Zhejiang | Ningbo | CCTCC-SBW0195 | ZNH4 | *Trichoderma atroviride* | Viride |
| Coastal form soil, Ningbo, Zhejiang | Ningbo | CCTCC-SBW0173 | ZNCF4 | *Trichoderma harzianum* | Green/Harzianum |
| Beach water, Ningbo, Zhejiang | Ningbo | CCTCC-SBW0164 | ZNBW8 | *Trichoderma harzianum* | Green/Harzianum |
| Aquaculture form soil, Ningbo, Zhejiang | Ningbo | CCTCC-SBW0119 | ZNAF1 | *Trichoderma atroviride* | Viride |
| Coastal form soil, Ningbo, Zhejiang | Ningbo | CCTCC-SBW0167 | ZNCF11 | *Trichoderma atroviride* | Viride |
| Coastal form soil, Ningbo, Zhejiang | Ningbo | CCTCC-SBW0176 | ZNCFW5 | *Trichoderma atroviride* | Viride |
| Reservoir soil, Ningbo, Zhejiang | Ningbo | CCTCC-SBW0206 | ZNR2 | *Trichoderma atroviride* | Viride |
| Coastal form water, Ningbo, Zhejiang | Ningbo | CCTCC-SBW0175 | ZNCFW3 | *Trichoderma harzianum* | Green/Harzianum |
| Beach water, Ningbo, Zhejiang | Ningbo | CCTCC-SBW0163 | ZNBW7 | *Trichoderma harzianum* | Green/Harzianum |
| Aquaculture form soil, Ningbo, Zhejiang | Ningbo | CCTCC-SBW0134 | ZNAF19 | *Trichoderma atroviride* | Viride |
| Wetland soil, Ningbo, Zhejiang | Ningbo | CCTCC-SBW0221 | ZNWPL5 | *Trichoderma atroviride* | Viride |
| Wetland soil, Ningbo, Zhejiang | Ningbo | CCTCC-SBW0222 | ZNWPL6 | *Trichoderma atroviride* | Viride |
| Aquaculture form soil, Ningbo, Zhejiang | Ningbo | CCTCC-SBW0124 | ZNAF12 | *Trichoderma aureoviride* | Green |
| Reservoir soil, Ningbo, Zhejiang | Ningbo | CCTCC-SBW0200 | ZNR10 | *Trichoderma atroviride* | Viride |
| Beach soil, Ningbo, Zhejiang | Ningbo | CCTCC-SBW0150 | ZNB5 | *Trichoderma atroviride* | Viride |
| Reservoir soil, Ningbo, Zhejiang | Ningbo | CCTCC-SBW0209 | ZNR9 | *Trichoderma atroviride* | Viride |
| Aquaculture form soil, Ningbo, Zhejiang | Ningbo | CCTCC-SBW0125 | ZNAF13 | *Trichoderma atroviride* | Viride |
| Aquaculture form soil, Ningbo, Zhejiang | Ningbo | CCTCC-SBW0121 | ZNAF10 | *Trichoderma harzianum* | Green/Harzianum |
| Reservoir soil, Ningbo, Zhejiang | Ningbo | CCTCC-SBW0201 | ZNR11 | *Trichoderma atroviride* | Viride |
| Wetland soil, Ningbo, Zhejiang | Ningbo | CCTCC-SBW0213 | ZNWPL12 | *Trichoderma atroviride* | Viride |
| Wetland soil, Ningbo, Zhejiang | Ningbo | CCTCC-SBW0214 | ZNWPL13 | *Trichoderma atroviride* | Viride |
| Wetland soil, Ningbo, Zhejiang | Ningbo | CCTCC-SBW0215 | ZNWPL14 | *Trichoderma atroviride* | Viride |
| Aquaculture form soil, Ningbo, Zhejiang | Ningbo | CCTCC-SBW0123 | ZNAF11 | *Trichoderma harzianum* | Green/Harzianum |
| Harbor soil, Ningbo, Zhejiang | Ningbo | CCTCC-SBW0194 | ZNH3 | *Trichoderma aureoviride* | Green |
| Harbor soil, Ningbo, Zhejiang | Ningbo | CCTCC-SBW0189 | ZNH11 | *Trichoderma aureoviride* | Green |
| Aquaculture form soil, Ningbo, Zhejiang | Ningbo | CCTCC-SBW0142 | ZNAF27 | *Trichoderma atroviride* | Viride |
| Wetland soil, Ningbo, Zhejiang | Ningbo | CCTCC-SBW0219 | ZNWPL18 | *Trichoderma atroviride* | Viride |
| Wetland soil, Ningbo, Zhejiang | Ningbo | CCTCC-SBW0216 | ZNWPL15 | *Trichoderma atroviride* | Viride |
| Aquaculture form soil, Ningbo, Zhejiang | Ningbo | CCTCC-SBW0120 | ZNAF1 | *Trichoderma atroviride* | Viride |
| Coastal form soil, Ningbo, Zhejiang | Ningbo | CCTCC-SBW0165 | ZNCF1 | *Trichoderma atroviride* | Viride |
| Coastal form soil, Ningbo, Zhejiang | Ningbo | CCTCC-SBW0174 | ZNCF6 | *Trichoderma atroviride* | Viride |
| Wetland soil, Ningbo, Zhejiang | Ningbo | CCTCC-SBW0218 | ZNWPL17 | *Trichoderma atroviride* | Viride |
| Aquaculture form soil, Ningbo, Zhejiang | Ningbo | CCTCC-SBW0130 | ZNAF16 | *Trichoderma atroviride* | Viride |
| Beach soil, Ningbo, Zhejiang | Ningbo | CCTCC-SBW0151 | ZNB6 | *Trichoderma atroviride* | Viride |
| Aquaculture form soil, Ningbo, Zhejiang | Ningbo | CCTCC-SBW0143 | ZNAF28 | *Trichoderma atroviride* | Viride |
| Aquaculture form soil, Ningbo, Zhejiang | Ningbo | CCTCC-SBW0132 | ZNAF17 | *Trichoderma atroviride* | Viride |
| Aquaculture form soil, Ningbo, Zhejiang | Ningbo | CCTCC-SBW0127 | ZNAF14 | *Trichoderma atroviride* | Viride |
| Aquaculture form soil, Ningbo, Zhejiang | Ningbo | CCTCC-SBW0131 | ZNAF16 | *Trichoderma atroviride* | Viride |
| Wetland soil, Ningbo, Zhejiang | Ningbo | CCTCC-SBW0210 | ZNWPL1 | *Trichoderma atroviride* | Viride |
| Harbor soil, Ningbo, Zhejiang | Ningbo | CCTCC-SBW0193 | ZNH2 | *Trichoderma atroviride* | Viride |
| Estuary soil, Ningbo, Zhejiang | Ningbo | CCTCC-SBW0182 | ZNE6 | *Trichoderma harzianum* | Green/Harzianum |
| Harbor soil, Ningbo, Zhejiang | Ningbo | CCTCC-SBW0197 | ZNH6 | *Trichoderma atroviride* | Viride |
| Beach water, Ningbo, Zhejiang | Ningbo | CCTCC-SBW0160 | ZNBW3 | *Trichoderma harzianum* | Green/Harzianum |
| Coastal form soil, Ningbo, Zhejiang | Ningbo | CCTCC-SBW0166 | ZNCF10 | *Trichoderma atroviride* | Viride |
| Aquaculture form soil, Ningbo, Zhejiang | Ningbo | CCTCC-SBW0148 | ZNAF9 | *Trichoderma atroviride* | Viride |
| Harbor soil, Ningbo, Zhejiang | Ningbo | CCTCC-SBW0187 | ZNH10 | *Trichoderma atroviride* | Viride |
| Wetland soil, Ningbo, Zhejiang | Ningbo | CCTCC-SBW0211 | ZNWPL10 | *Trichoderma atroviride* | Viride |
| Wetland soil, Ningbo, Zhejiang | Ningbo | CCTCC-SBW0223 | ZNWPL7 | *Trichoderma atroviride* | Viride |
| Wetland soil, Ningbo, Zhejiang | Ningbo | CCTCC-SBW0204 | ZNR18 | *Trichoderma atroviride* | Viride |
| Reservoir soil, Ningbo, Zhejiang | Ningbo | CCTCC-SBW0207 | ZNR20 | *Trichoderma harzianum* | Green/Harzianum |
| Reservoir soil, Ningbo, Zhejiang | Ningbo | CCTCC-SBW0220 | ZNWPL4 | *Trichoderma atroviride* | Viride |
| Wetland soil, Ningbo, Zhejiang | Ningbo | CCTCC-SBW0133 | ZNAF18 | *Trichoderma atroviride* | Viride |
| Aquaculture form soil, Ningbo, Zhejiang | Ningbo | CCTCC-SBW0159 | ZNBW2 | *Trichoderma atroviride* | Viride |
| Beach water, Ningbo, Zhejiang | Ningbo | CCTCC-SBW0180 | ZNE3 | *Trichoderma harzianum* | Green/Harzianum |
| Estuary soil, Ningbo, Zhejiang | Ningbo | CCTCC-SBW0225 | ZNWPL9 | *Trichoderma harzianum* | Green/Harzianum |
| Wetland soil, Ningbo, Zhejiang | Ningbo | CCTCC-SBW0161 | ZNBW3 | *Trichoderma harzianum* | Green/Harzianum |
| Beach water, Ningbo, Zhejiang | Ningbo | CCTCC-SBW0147 | ZNAF5 | *Trichoderma atroviride* | Viride |
| Aquaculture form soil, Ningbo, Zhejiang | Ningbo | CCTCC-SBW0128 | ZNAF14 | *Trichoderma atroviride* | Viride |
| Aquaculture form soil, Ningbo, Zhejiang | Ningbo | CCTCC-SBW0126 | ZNAF13 | *Trichoderma atroviride* | Viride |
| Aquaculture form soil, Ningbo, Zhejiang | Ningbo | CCTCC-SBW0126 | ZNAF13 | *Trichoderma atroviride* | Viride |
| Aquaculture form soil, Ningbo, Zhejiang | Ningbo | CCTCC-SBW0141 | ZNAF24 | *Trichoderma atroviride* | Viride |
| Estuary soil, Ningbo, Zhejiang | Ningbo | CCTCC-SBW0186 | ZNEF21 | *Trichoderma atroviride* | Viride |
| Beach soil, Ningbo, Zhejiang | Ningbo | CCTCC-SBW0149 | ZNB4 | *Trichoderma harzianum* | Green/Harzianum |
| Beach water, Ningbo, Zhejiang | Ningbo | CCTCC-SBW0162 | ZNBW4 | *Trichoderma harzianum* | Green/Harzianum |
| Coastal form soil, Ningbo, Zhejiang | Ningbo | CCTCC-SBW0172 | ZNCF22 | *Trichoderma atroviride* | Viride |
| Coastal form soil, Ningbo, Zhejiang | Ningbo | CCTCC-SBW0168 | ZNCF14 | *Trichoderma atroviride* | Viride |
| Reservoir soil, Ningbo, Zhejiang | Ningbo | CCTCC-SBW0202 | ZNR14 | *Trichoderma atroviride* | Viride |
| Wetland soil, Ningbo, Zhejiang | Ningbo | CCTCC-SBW0212 | ZNWPL11 | *Trichoderma aureoviride* | Green |
| Harbor soil, Ningbo, Zhejiang | Ningbo | CCTCC-SBW0196 | ZNH5 | *Trichoderma atroviride* | Viride |
| Beach soil, Ningbo, Zhejiang | Ningbo | CCTCC-SBW0153 | ZNB8 | *Trichoderma atroviride* | Viride |
| Harbor soil, Ningbo, Zhejiang | Ningbo | CCTCC-SBW0198 | ZNH8 | *Trichoderma atroviride* | Viride |
| Aquaculture form soil, Ningbo, Zhejiang | Ningbo | CCTCC-SBW0140 | ZNAF23 | *Trichoderma atroviride* | Viride |
| Aquaculture form soil, Ningbo, Zhejiang | Ningbo | CCTCC-SBW0146 | ZNAF31 | *Trichoderma harzianum* | Green/Harzianum |
| Aquaculture form soil, Ningbo, Zhejiang | Ningbo | CCTCC-SBW0135 | ZNAF2 | *Trichoderma atroviride* | Viride |
| Harbor soil, Ningbo, Zhejiang | Ningbo | CCTCC-SBW0199 | ZNH9 | *Trichoderma atroviride* | Viride |
| Aquaculture form soil, Ningbo, Zhejiang | Ningbo | CCTCC-SBW0138 | ZNAF21 | *Trichoderma atroviride* | Viride |
| Reservoir soil, Ningbo, Zhejiang | Ningbo | CCTCC-SBW0203 | ZNR15 | *Trichoderma atroviride* | Viride |
| Beach soil, Ningbo, Zhejiang | Ningbo | CCTCC-SBW0152 | ZNB7 | *Trichoderma harzianum* | Green/Harzianum |
| Aquaculture form soil, Ningbo, Zhejiang | Ningbo | CCTCC-SBW0122 | ZNAF10 | *Trichoderma aureoviride* | Green |
| Beach water, Ningbo, Zhejiang | Ningbo | CCTCC-SBW0157 | ZNBW14 | *Trichoderma aureoviride* | Green |
| Aquaculture form, Lianyungang, Jiangsu | Lianyungang | CCTCC-SBW0072 | LEAF6 | *Trichoderma atroviride* | Viride |
| Beach soil, Lianyungang, Jiangsu | Lianyungang | CCTCC-SBW0073 | LEBE1 | *Trichoderma atroviride* | Viride |
| Aquaculture form, Lianyungang, Jiangsu | Lianyungang | CCTCC-SBW0067 | LEAF1 | *Trichoderma atroviride* | Viride |
| Beach soil, Lianyungang, Jiangsu | Lianyungang | CCTCC-SBW0074 | LEBE2 | *Trichoderma atroviride* | Viride |
| Aquaculture form, Lianyungang, Jiangsu | Lianyungang | CCTCC-SBW0070 | LEAF4 | *Trichoderma atroviride* | Viride |
| Aquaculture form, Lianyungang, Jiangsu | Lianyungang | CCTCC-SBW0069 | LEAF3 | *Trichoderma atroviride* | Viride |
| Aquaculture form, Lianyungang, Jiangsu | Lianyungang | CCTCC-SBW0068 | LEAF2 | *Trichoderma atroviride* | Viride |
| Aquaculture form, Lianyungang, Jiangsu | Lianyungang | CCTCC-SBW0071 | LEAF5 | *Trichoderma atroviride* | Viride |
| Wetland soil, Zhuhai, Guangdong | Zhuhai | CCTCC-SBW0116 | ZMWE | *Trichoderma atroviride* | Viride |
| Wetland soil, Zhuhai, Guangdong | Zhuhai | CCTCC-SBW0115 | ZWATE | *Trichoderma atroviride* | Viride |
| Wetland soil, Behai, Guangxi | Behai | CCTCC-SBW0001 | BMWT1 | *Trichoderma atroviride* | Viride |
| Wetland soil, Behai, Guangxi | Behai | CCTCC-SBW0002 | BMWT2 | *Trichoderma atroviride* | Viride |
| Wetland soil, Behai, Guangxi | Behai | CCTCC-SBW0003 | BMWT5 | *Trichoderma atroviride* | Viride |
| Wetland soil, Fuzhou, Fujian | Fuzhou | CCTCC-SBW0062 | FWT3 | *Trichoderma atroviride* | Viride |
| Wetland soil, Fuzhou, Fujian | Fuzhou | CCTCC-SBW0065 | FWT6 | *Trichoderma atroviride* | Viride |
| Wetland soil, Fuzhou, Fujian | Fuzhou | CCTCC-SBW0063 | FWT4 | *Trichoderma harzianum* | Green/Harzianum |
| Wetland soil, Zhuhai, Guangdong | Zhuhai | CCTCC-SBW0117 | ZMWE4 | *Trichoderma atroviride* | Viride |
| Wetland soil, Zhuhai, Guangdong | Zhuhai | CCTCC-SBW0118 | ZMWE5 | *Trichoderma harzianum* | Green/Harzianum |
| Mangroves, Wetland soil, Zhuhai, Guangdong | Zhuhai | CCTCC-SBW0110 | ZMWE1 | *Trichoderma atroviride* | Viride |
| Wetland soil, Shantou, Guangdong | Shantou | CCTCC-SBW0081 | STWT4 | *Trichoderma atroviride* | Viride |
| Wetland soil, Shantou, Guangdong | Shantou | CCTCC-SBW0075 | SMWE6 | *Trichoderma atroviride* | Viride |
| Wetland soil, Fuzhou, Fujian | Fuzhou | CCTCC-SBW0059 | FWE1 | *Trichoderma atroviride* | Viride |
| Wetland soil, Fuzhou, Fujian | Fuzhou | CCTCC-SBW0064 | FWT5 | *Trichoderma atroviride* | Viride |
| Wetland soil, Fuzhou, Fujian | Fuzhou | CCTCC-SBW0061 | FWT1 | *Trichoderma atroviride* | Viride |
| Wetland soil, Fuzhou, Fujian | Fuzhou | CCTCC-SBW0066 | FWT7 | *Trichoderma harzianum* | Green/Harzianum |
| Wetland soil, Shantou, Guangdong | Shantou | CCTCC-SBW0082 | STWT5 | *Trichoderma atroviride* | Viride |
| Wetland soil, Shantou, Guangdong | Shantou | CCTCC-SBW0087 | STWT9 | *Trichoderma atroviride* | Viride |
| Wetland soil, Shantou, Guangdong | Shantou | CCTCC-SBW0078 | STWT12 | *Trichoderma atroviride* | Viride |
| Wetland soil, Shantou, Guangdong | Shantou | CCTCC-SBW0079 | STWT2 | *Trichoderma harzianum* | Green/Harzianum |
| Wetland soil, Shantou, Guangdong | Shantou | CCTCC-SBW0077 | STWT1 | *Trichoderma harzianum* | Green/Harzianum |
| Wetland soil, Shantou, Guangdong | Shantou | CCTCC-SBW0085 | STWT6 | *Trichoderma harzianum* | Green/Harzianum |
| Wetland soil, Shantou, Guangdong | Shantou | CCTCC-SBW0086 | STWT7 | *Trichoderma atroviride* | Viride |
| Wetland soil, Shantou, Guangdong | Shantou | CCTCC-SBW0083 | STWT5 | *Trichoderma atroviride* | Viride |
| Wetland soil, Fuzhou, Fujian | Fuzhou | CCTCC-SBW0060 | FWE2 | *Trichoderma atroviride* | Viride |
| Mangroves, Wetland soil, Zhuhai, Guangdong | Zhuhai | CCTCC-SBW0111 | ZMWE2 | *Trichoderma atroviride* | Viride |
| Mangroves, Wetland soil, Zhuhai, Guangdong | Zhuhai | CCTCC-SBW0112 | ZWATE1 | *Trichoderma atroviride* | Viride |
| Mangroves, Wetland water, Zhuhai, Guangdong | Zhuhai | CCTCC-SBW0113 | ZWATE9 | *Trichoderma atroviride* | Viride |
| Wetland soil, Xixa park, Chongming island, Shanghai | Shanghai | CCTCC-SBW0026 | SCXIX1 | *Trichoderma harzianum* | Green/Harzianum |
| Wetland soil, Xixa park, Chongming island, Shanghai | Shanghai | CCTCC-SBW0037 | SCXIX2 | *Trichoderma harzianum* | Green/Harzianum |
| Wetland soil, Xixa park, Chongming island, Shanghai | Shanghai | CCTCC-SBW0043 | SCXIX3 | *Trichoderma atroviride* | Viride |
| Wetland soil, Xixa park, Chongming island, Shanghai | Shanghai | CCTCC-SBW0044 | SCXIX4 | *Trichoderma atroviride* | Viride |
| Wetland soil, Xixa park, Chongming island, Shanghai | Shanghai | CCTCC-SBW0045 | SCXIX5 | *Trichoderma atroviride* | Viride |
| Wetland soil, Xixa park, Chongming island, Shanghai | Shanghai | CCTCC-SBW0046 | SCXIX6 | *Trichoderma aureoviride* | Green |
| Wetland soil, Xixa park, Chongming island, Shanghai | Shanghai | CCTCC-SBW0047 | SCXIX7 | *Trichoderma atroviride* | Viride |
| Wetland soil, Xixa park, Chongming island, Shanghai | Shanghai | CCTCC-SBW0048 | SCXIX8 | *Trichoderma atroviride* | Viride |
| Wetland soil, Xixa park, Chongming island, Shanghai | Shanghai | CCTCC-SBW0049 | SCXIX9 | *Trichoderma atroviride* | Viride |
| Wetland soil, Xixa park, Chongming island, Shanghai | Shanghai | CCTCC-SBW0027 | SCXIX10 | *Trichoderma atroviride* | Viride |
| Wetland soil, Xixa park, Chongming island, Shanghai | Shanghai | CCTCC-SBW0028 | SCXIX11 | *Trichoderma harzianum* | Green/Harzianum |
| Wetland soil, Xixa park, Chongming island, Shanghai | Shanghai | CCTCC-SBW0029 | SCXIX12 | *Trichoderma atroviride* | Viride |
| Wetland soil, Xixa park, Chongming island, Shanghai | Shanghai | CCTCC-SBW0030 | SCXIX13 | *Trichoderma atroviride* | Viride |
| Wetland soil, Xixa park, Chongming island, Shanghai | Shanghai | CCTCC-SBW0031 | SCXIX14 | *Trichoderma atroviride* | Viride |
| Wetland soil, Xixa park, Chongming island, Shanghai | Shanghai | CCTCC-SBW0032 | SCXIX15 | *Trichoderma atroviride* | Viride |
| Wetland soil, Xixa park, Chongming island, Shanghai | Shanghai | CCTCC-SBW0033 | SCXIX16 | *Trichoderma harzianum* | Green/Harzianum |
| Wetland soil, Xixa park, Chongming island, Shanghai | Shanghai | CCTCC-SBW0034 | SCXIX17 | *Trichoderma aureoviride* | Green |
| Wetland soil, Xixa park, Chongming island, Shanghai | Shanghai | CCTCC-SBW0035 | SCXIX18 | *Trichoderma aureoviride* | Green |
| Wetland soil, Xixa park, Chongming island, Shanghai | Shanghai | CCTCC-SBW0036 | SCXIX19 | *Trichoderma atroviride* | Viride |
| Wetland soil, Xixa park, Chongming island, Shanghai | Shanghai | CCTCC-SBW0038 | SCXIX20 | *Trichoderma atroviride* | Viride |
| Wetland soil, Xixa park, Chongming island, Shanghai | Shanghai | CCTCC-SBW0039 | SCXIX21 | *Trichoderma atroviride* | Viride |
| Wetland soil, Xixa park, Chongming island, Shanghai | Shanghai | CCTCC-SBW0040 | SCXIX22 | *Trichoderma atroviride* | Viride |
| Wetland soil, Xixa park, Chongming island, Shanghai | Shanghai | CCTCC-SBW0041 | SCXIX23 | *Trichoderma atroviride* | Viride |
| Wetland soil, Xixa park, Chongming island, Shanghai | Shanghai | CCTCC-SBW0042 | SCXIX24 | *Trichoderma atroviride* | Viride |
| Wetland soil, Xixa park, Chongming island, Shanghai | Shanghai | CCTCC-SBW0042 | SCXIX25 | *Trichoderma atroviride* | Viride |
