# Supplementary material for: Biodiversity of Trichoderma Community in the Tidal Flats and Wetland of Southeastern China
Source: PLoS One. 2016 Dec 21;11(12):e0168020. doi: 10.1371/journal.pone.0168020 (PMC5176281; doi:10.1371/journal.pone.0168020)
Supplement: S3 Table — 6 univariate 2- way ANOVAs used followed by multiple comparison teats (SNK and Tukey’s) to examine effects of genotype species and season on seven biochemical response variables. ** = p < 0.01;* = p < 0.05; NS = Not Significant. (DOC) [file pone.0168020.s006.doc]

**S3 Table Physical and chemical characteristics and *Trichoderma* counts ( CFU ) of soil in sampling stations in five soil depths in two seasons. 6 univariate 2- way ANOVAs used followed by multiple comparison teats (SNK and Tukey’s) to examine effects of genotype species and season on seven biochemical response variables. ** = p < 0.01;* = p < 0.05; NS= Not Significant**

| **Source** | Temperature (°C) | Salinity (ppt) | Eh(mV) | pH | Silt (%) | Clay (%) | Sand (%) | TOC (mgC.g^-1^ soil) | *Trichoderma* ( CFU x 10^4^.g-1 of soil) |
| --- | --- | --- | --- | --- | --- | --- | --- | --- | --- |
| **Sampling stations** |  |  |  |  |  |  |  |  |  |
| Beihai | 23.31±0.28^d^ | 25.92±0.93^c^ | -148.82±12.5^a^ | 7.92±0.1^b^ | 24.58±1.29^d^ | 25.60±1.89^c^ | 50.89±3.13^a^ | 3.19±0.37^c^ | 0.180±0.04^a^ |
| Chongming | 15.57±0.32^a^ | 9.67±10.7^b^ | -43.41±14.4^c^ | 8.38±0.1^c^ | 4.99±1.49^a^ | 3.04±2.18^a^ | 91.97±3.61^c^ | 2.09±0.42^b^ | 0.240±0.05^a^ |
| Fengxian | 16.86±0.32^b^ | 0.86±1.07^a^ | -154.06±14.4^a^ | 8.61±0.1^c^ | 6.50±1.26^a^ | 4.73±2.18^a^ | 89.77±3.61^c^ | 5.38±0.42^d^ | 0.100±0.05^a^ |
| Fuzhou | 24.59±0.32^e^ | 22.93±1.07^c^ | -17.46±14.4^c^ | 8.25±0.1^c^ | 25.18±1.45^d^ | 25.11±2.18^c^ | 50.20±3.61^a^ | 1.03±0.42^a^ | 0.160±0.05^a^ |
| Hangzhou | 24.51±0.25^e^ | 3.26±0.83^a^ | -64.62±11.1^c^ | 7.95±0.09^b^ | 7.45±1.15^a^ | 7.16±1.69^b^ | 85.83±2.80^c^ | 1.77±0.33^a^ | 0.156±0.04^a^ |
| Lianyugang | 23.65±0.25^d^ | 8.99±0.83^b^ | -58.82±11.1^b^ | 8.00±0.09^b^ | 18.03±1.16^c^ | 25.70±1.69^c^ | 57.51±2.80^a^ | 2.21±0.33^b^ | 0.164±0.04^a^ |
| Ningbo | 25.54±0.21^f^ | 3.83±0.71^a^ | -97.51±9.5^b^ | 8.06±0.08^b^ | 16.48±0.98^c^ | 20.01±1.44^c^ | 64.58±2.38^b^ | 2.94±0.28^b^ | 0.480±0.03^c^ |
| Shantou | 23.18±0.34^d^ | 11.06±1.13^b^ | -80.22±15.2^b^ | 7.55±0.12^a^ | 10.13±1.57^b^ | 9.38±2.30^b^ | 80.54±3.81^c^ | 3.34±0.45^c^ | 0.215±0.05^b^ |
| Zhuhai | 20.60±0.21^c^ | 25.05±0.65^c^ | -37.47±8.3^c^ | 7.77±0.07^a^ | 5.75±0.91^a^ | 0.01±1.34^a^ | 94.19±2.21^d^ | 1.65±0.26^a^ | 0.288±0.03^b^ |
| **Soil depth (cm)** |  |  |  |  |  |  |  |  |  |
| 0-20 | 22.96±0.21^b^ | 12.51±0.69 | 44.27±9.3^d^ | 8.37±0.07^c^ | 13.23±0.96 | 13.38±1.41 | 73.98±2.31 | 2.94±0.27 | 0.685±0.03^c^ |
| 20-40 | 22.40±0.56^b^ | 12.76±0.69 | 4.44±9.3^c^ | 8.21±0.07^c^ | 13.33±0.92 | 13.42±1.25 | 73.92±2.35 | 2.74±0.27 | 0.309±0.02^b^ |
| 40-60 | 21.84±1.21^a^ | 12.73±0.70 | -1.06±9.4^c^ | 8.08±0.08^b^ | 13.39±0.85 | 14.12±1.36 | 73.24±2.45 | 2.81±0.28 | 0.092±0.03^a^ |
| 60-80 | 21.39±2.3^a^ | 11.85±0.69 | -146.66±9.3^b^ | 7.84±0.07^a^ | 13.14±0.12 | 13.18±1.84 | 73.99±2.26 | 2.44±0.27 | 0.006±0.03^a^ |
| 80-100 | 21.22±1.5^a^ | 12.22±0.69 | -187.06±9.3^a^ | 7.80±0.07^a^ | 13.26±0.63 | 13.23±1.56 | 74.19±2.45 | 2.15±0.27 | 0.004±0.01^a^ |
| **Season of analysis** |  |  |  |  |  |  |  |  |  |
| Spring | 17.93±0.13^a^ | 9.32±0.44^b^ | -89.36±5.94^a^ | 8.18±0.5^a^ | 9.98±0.61^a^ | 0.04±0.90^a^ | 89.97±1.49^b^ | 2.60±0.17 | 0.17±0.02^a^ |
| Summer | 20.95±0.14^b^ | 15.44±0.4^a^ | -66.93±5.89^b^ | 7.95±0.5^b^ | 16.48±0.61^b^ | 26.58±0.89^b^ | 58.12±1.47^a^ | 2.63±0.15 | 0.26±0.02^b^ |
| Station | ** | ** | ** | ** | ** | ** | ** | ** | ** |
| Depth | ** | NS | * | ** | NS | NS | NS | NS | ** |
| Season | ** | ** | ** | ** | ** | ** | ** | NS | ** |
|  |  |  |  |  |  |  |  |  |  |
