# Supplementary material for: Biodiversity of Trichoderma Community in the Tidal Flats and Wetland of Southeastern China
Source: PLoS One. 2016 Dec 21;11(12):e0168020. doi: 10.1371/journal.pone.0168020 (PMC5176281; doi:10.1371/journal.pone.0168020)
Supplement: S4 Table — (DOC) [file pone.0168020.s007.doc]

**S4 Table *Trichoderma* counts in relation to varied physical- chemical factors**

|  | Temperature (°C) | Salinity (ppt) | Eh(mV) | pH | Silt (%) | Clay (%) | Sand (%) | TOC (mgC.g^-1^ soil) | *Trichoderma* ( CFU x 10^4^.g-1 of soil) |
| --- | --- | --- | --- | --- | --- | --- | --- | --- | --- |
| Temperature (°C) | 1 |  |  |  |  |  |  |  |  |
| Salinity (ppt) | .148^**^ | 1 |  |  |  |  |  |  |  |
| Eh(mV) | .231^**^ | .094 | 1 |  |  |  |  |  |  |
| pH | -.244^**^ | -.120^*^ | -.016 | 1 |  |  |  |  |  |
| Silt (%) | .150^**^ | .135^**^ | -.060 | .143^**^ | 1 |  |  |  |  |
| Clay (%) | .373^**^ | .100^*^ | .009 | .048 | .887^**^ | 1 |  |  |  |
| Sand (%) | -.293^**^ | -.122^*^ | .018 | -.088 | -.955^**^ | -.981^**^ | 1 |  |  |
| TOC (mgC.g^-1^ soil) | -.154^**^ | -.201^**^ | -.136^**^ | .107^*^ | .127^*^ | .108^*^ | -.111^*^ | 1 |  |
| *Trichoderma* ( CFU x 10^4^.g-1 of soil) | .135^**^ | .017 | .366^**^ | .123^*^ | -.015 | -.010 | .012 | .024 | 1 |
|  |  |  |  |  |  |  |  |  |  |

**. Correlation is significant at the 0.01 level (2-tailed). *. Correlation is significant at the 0.05 level (2-tailed).
