# Supplementary material for: Biodiversity of Trichoderma Community in the Tidal Flats and Wetland of Southeastern China
Source: PLoS One. 2016 Dec 21;11(12):e0168020. doi: 10.1371/journal.pone.0168020 (PMC5176281; doi:10.1371/journal.pone.0168020)
Supplement: S5 Table — (DOC) [file pone.0168020.s008.doc]

**S5 Table** *Trichoderma* species richness index

| Samples | Chao | ace |
| --- | --- | --- |
| wt11 | 414.541 | 421.379 |
| wt12 | 326.517 | 337.358 |
| wt13 | 230.92 | 236.548 |
| wt14 | 569.51 | 580.328 |
| wt15 | 437.859 | 452.002 |
| wt16 | 555.896 | 557.246 |
| wt17 | 342.278 | 351.204 |
| wt18 | 58.1538 | 59.995 |
| wt19 | 648.377 | 666.065 |
| wt20 | 599.822 | 613.96 |
