# Supplementary material for: Biodiversity of Trichoderma Community in the Tidal Flats and Wetland of Southeastern China
Source: PLoS One. 2016 Dec 21;11(12):e0168020. doi: 10.1371/journal.pone.0168020 (PMC5176281; doi:10.1371/journal.pone.0168020)
Supplement: S6 Table — P values based on 999 permutations. (DOC) [file pone.0168020.s009.doc]

**S6 Table** Redundancy discriminant analysis (RDA) of the distribution of *Trichoderma* species with environmental variables of the study stations significant codes: '*' 0.05 ' ‘NS’ not significant. P values based on 999 permutations.

|  | RDA1 | RDA2 | r2 | Pr(>r) |
| --- | --- | --- | --- | --- |
| Temperature (°C) | -0.76404 | 0.64517 | 0.2313 | 0.0331^*^ |
| Salinity (ppt) | 0.25584 | -0.96672 | 0.0097 | 0.94^NS^ |
| Eh(mV) | -0.71694 | 0.69714 | 0.0831 | 0.0563^*^ |
| pH | 0.80426 | -0.59427 | 0.1835 | 0.0481^*^ |
| Silt (%) | 0.653 | 0.75735 | 0.0793 | 0.788^NS^ |
| Clay (%) | 0.20957 | 0.97779 | 0.1935 | 0.473^NS^ |
| Sand (%) | -0.36683 | -0.93029 | 0.1327 | 0.647^NS^ |
| TOC (mgC.g^-1^ soil) | 0.81694 | -0.57672 | 0.1113 | 0.664^NS^ |
|  |  |  |  |  |
